# Supplementary material for: eNOS-dependent S-nitrosylation of the NF-κB subunit p65 has neuroprotective effects
Source: Cell Death Dis. 2021 Jan 4;12(1):4. doi: 10.1038/s41419-020-03338-4 (PMC7790835; doi:10.1038/s41419-020-03338-4)
Supplement: Supplementary file 1 — Supplemental Figures and Tables [file 41419_2020_3338_MOESM1_ESM.pdf]

SUPPLEMENTAL FIGURES

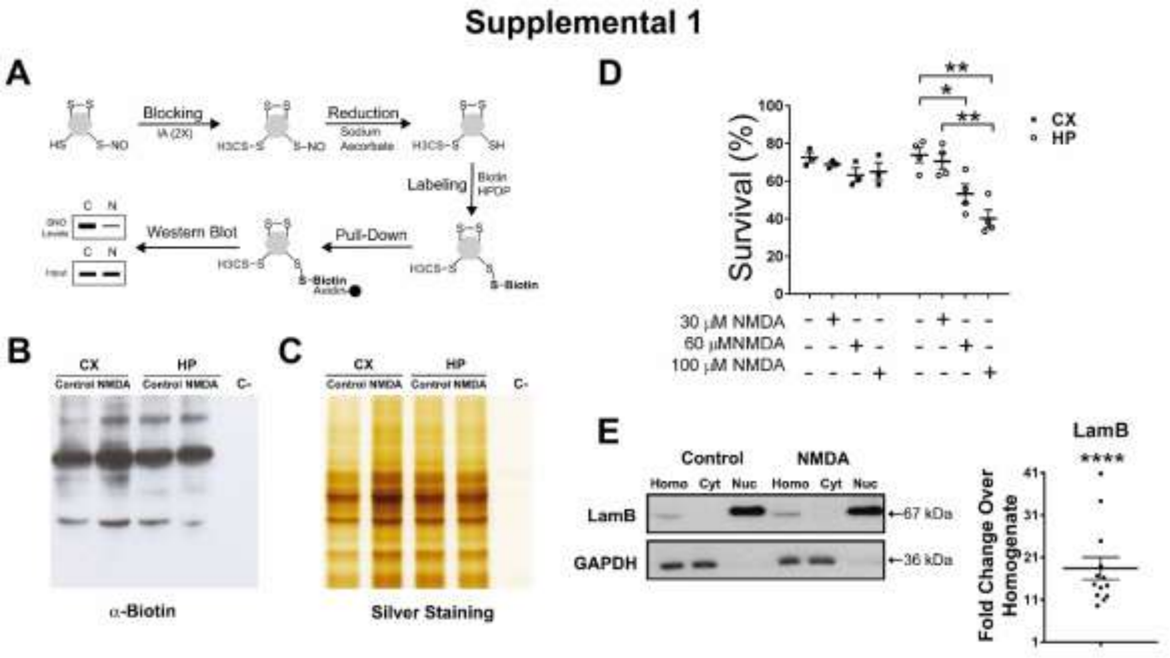

Supplemental Figure S1. Methodological aspects: A to C: Biotin switch assay in neuronal cell cultures, D: NMDA doses and cell death in cell cultures, E: obtention of nuclear fractions.

A) Biotin switch assay. Free –SH groups were blocked in 1 mg of starting material 2 times with 100 mM iodoacetamide for one hour at room temperature, followed by reduction of S-nitrosylated cysteines with 100 mM sodium ascorbate and subsequent labeling them with 300 µM biotin HPDP for one hour. Biotinylated proteins were considered as formerly S-nitrosylated ones and pulled down by streptavidin-beads. The figure was based on Forrester et al. [17]. In the negative control (C-), sodium ascorbate was omitted and thus, finally no biotinylated proteins are expected. **B) Detection of the biotinylated proteins** by Western blot using an anti-biotin antibody in cortical and hippocampal cultures, showing correct -SH group blocking and biotin labeling. **C) Silver staining of biotinylated**

proteins captured by streptavidin beads and separated in a 10 % SDS–PAGE. **D) Dose response curve for NMDA.** Cell viability was assessed at different NMDA concentrations, applied for one hour to measure cell death with the Trypan exclusion test 24 hours later. Hippocampal (HP) or cortical (CX) cultures were studied. 30 µM NMDA stimulation induces no significant cell death, analyzed in n= 3 to 4 independent experiment. Statistical significance was assessed by One-way ANOVA followed by Bonferroni post-test. **$p<0.01$; *$p<0.05$. **E) Nuclear fractionation.** Approval of sufficient purification of prepared nuclear fractions by the nuclear marker Laminin B1. Representative Western blot of total levels of Lamin B1 and GAPDH (as cytoplasmic marker) in subcellular fractions: homogenate, and the cytoplasmic and nuclear fractions of control and 30 µM NMDA stimulated neuronal cultures. An enrichment of Lamin B1 and absence of GAPDH in the nuclear fraction is observed in each case. The densitometric quantification (fold change in the nuclear fraction over homogenate) of Lamin B1 is shown (n=13 biological replicates). **** $p<0.0001$ by two-tailed t-test. Homo=homogenate; Cyt= cytoplasmic fraction; Nuc= nuclear fraction.

Supplemental 2

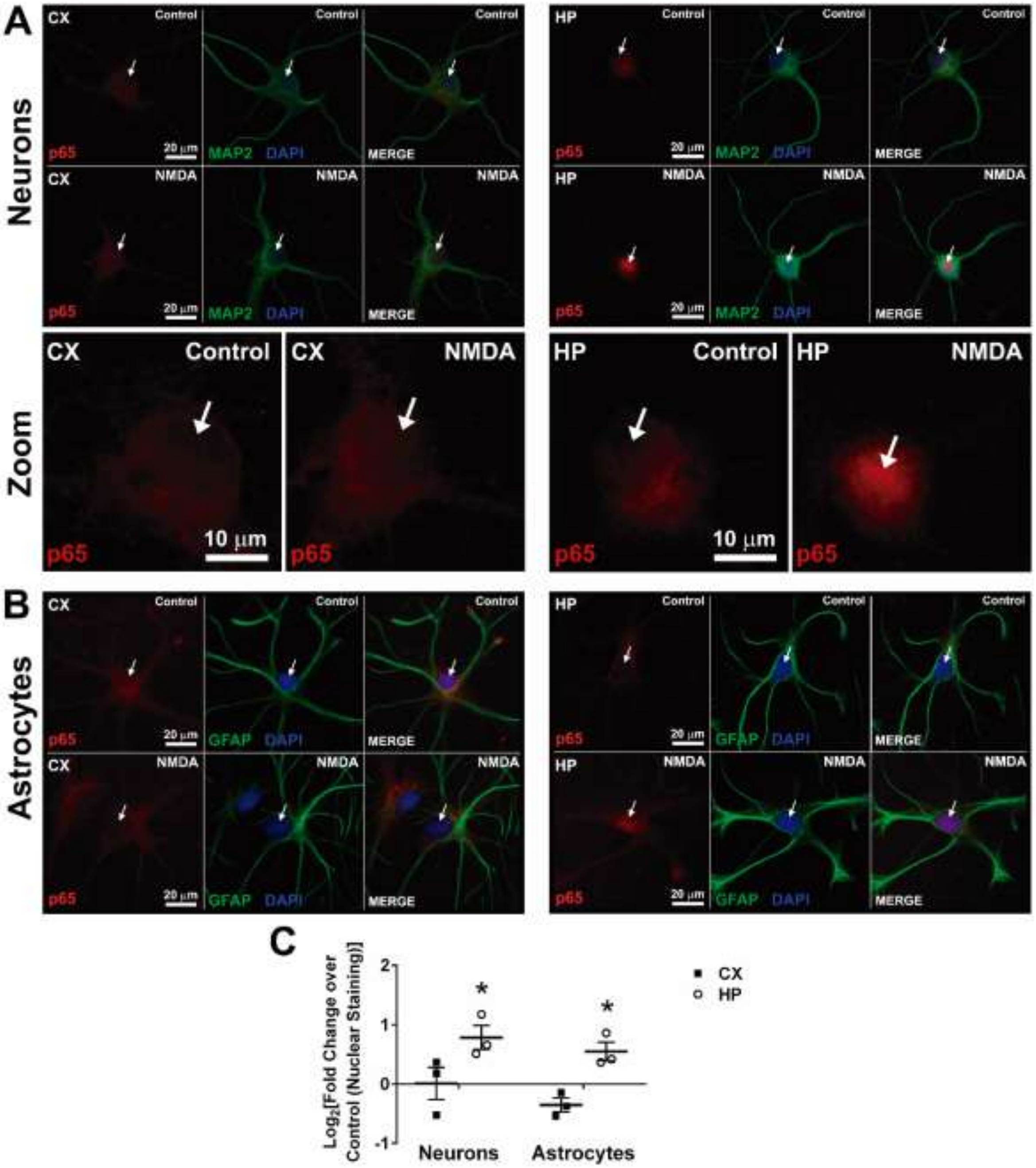

Supplemental Figure S2. 30 µM **NMDA induces the nuclear translocation of p65 (NF-κB) in hippocampal but not in cortical cultures.** We detected p65 in DAPI-stained nuclei of neurons (labelled with an antibody against microtubule associated protein 2, MAP2) or astrocytes (labelled with an antibody against glial fibrillary associated protein, GFAP). Neurons (top panel) and Astrocytes (Bottom panel) of cortical (left) and hippocampal (right) cultures stimulated with 30 µM NMDA for 60 minutes, were stained with antibodies against p65 (red), MAP2 (neurons, green) or GFAP (astrocytes, green). Cell nuclei were stained with DAPI (blue). The fluorescence intensity in the ROI (*i.e.*, the DAPI-positive region) was quantified. **A)** Representative images of a cortical (left) and hippocampal (right) neuronal cultures stained with p65, MAP2 and DAPI at two magnifications (calibration bars of 10 µm and 20 µM, respectively). ~~.~~ Arrows indicate cell nuclei recognized by DAPI staining. **B)** Representative images of a cortical (left) and hippocampal (right) astrocyte cultures stained with p65, GFAP and DAPI. **C)** Relative changes of p65 fluorescence intensity in the nuclei of neurons and astrocytes of cortical (CX) and hippocampal (HP) cultures when comparing the stimulated with the control (non-stimulated) condition. Results obtained in n=4 independent experiments. * $p<0.05$ by two-tailed t-test.

Supplemental 3

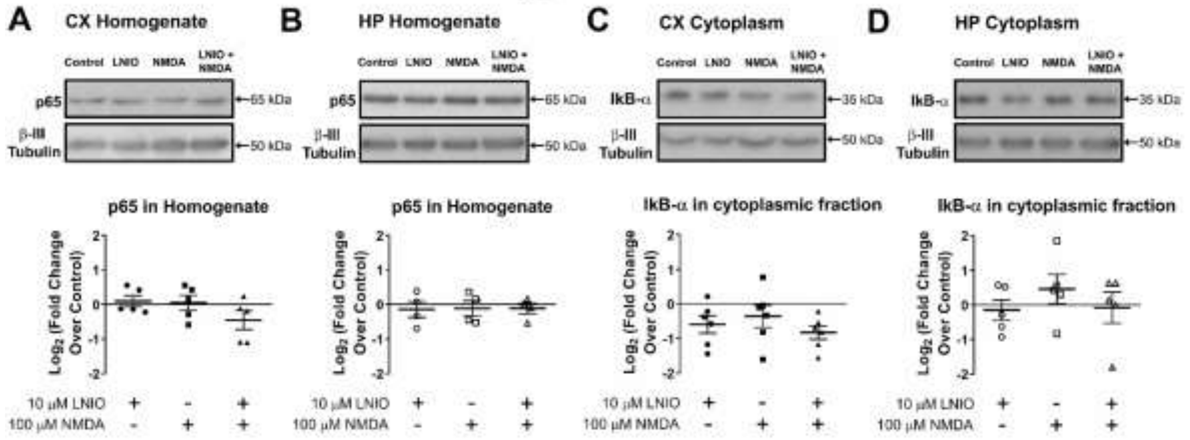

Supplemental Figure S3. The expression levels of NF-κB subunit p65 in homogenates and of IkB-α in cytoplasmic fractions remain constant in all conditions. A) and B) Representative Western blots and densitometric quantification of cytoplasmic content of IkB-α in cortical (A) and hippocampal (B) cultures stimulates with NMDA 100 µM in presence or absence of NO inhibitor LNIO (N5-(1-Iminoethyl)-L-ornithine). **C) and D)** Representative Wester blots and densitometric quantification of total content of p65 in cortical (C) and hippocampal (D) cultures stimulates with NMDA 100 µM in presence or absence of NO inhibitor LNIO. For each Western blot, equal quantities of protein were loaded, and β-III tubulin was used as loading control for cytoplasmic fraction and homogenate.

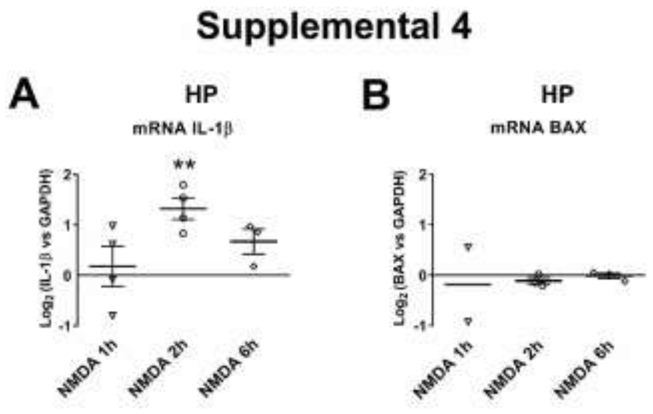

Supplemental Figure S4. Time course of IL-1β mRNA upregulation in response to NMDA in hippocampal cultures. A) and B) IL-1β mRNA (left) and BAX mRNA (right) measured by quantitative PCR in hippocampal cultures 1, 2 and 6 hours after 100 μM NMDA stimuli. Bar graphs show the mean ± SEM fold change normalized against GAPDH as reference. Data was obtained from 2 to 4 independent hippocampal cultures. Statistical significance was assessed by One-way ANOVA followed by Bonferroni post-test. $^{**}p<0.01$.

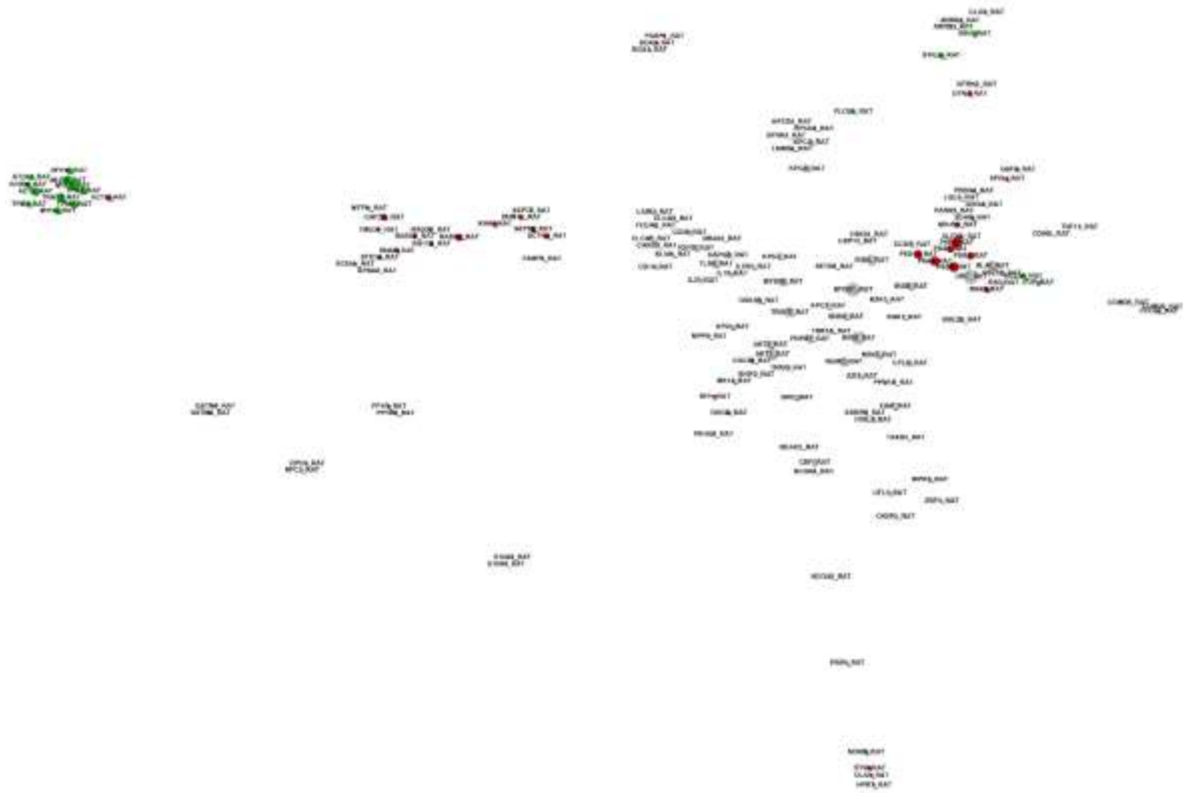

Supplemental Figure S5. The annotated Gene Ontology terms of biological processes were retrieved for the identified proteins that were exclusively detected in cortical (red) or hippocampal (green) proteomes and NF-κB related proteins (grey). In Figure 2 G the proteins are depicted as nodes with shared biological processes shown as edges connecting the nodes, forming a graph. To better display the similarity between the different protein groups a force field embedder was used to calculate a layout, thus depicting similar proteins closer to each other.

SUPPLEMENTAL TABLES

SUPPLEMENTAL TABLE 1

Figure 1C	Mean ± SE	n	p value		
HP Control vs HP 30 µM NMDA	1.00 vs 1.84 ± 0.37	5	> 0.05		
HP Control vs HP 100 µM NMDA	1.00 vs 2.07 ± 0.16	5	> 0.001		
CX Control vs CX 30 µM NMDA	1.00 vs 1.14 ± 0.17	9	n.s		
CX Control vs CX 100 µM NMDA	1.00 vs 1.26 ± 0.15	8	n.s		
**Figure 1D**	**Mean ± SE**	**n**	**p value**		
HP Control vs HP 30 µM NMDA	1.00 vs 0.75 ± 0.16	4	n.s		
HP Control vs HP 100 µM NMDA	1.00 vs 1.02 ± 0.21	4	n.s		
CX Control vs CX 30 µM NMDA	1.00 vs 0.79 ± 0.03	3	n.s		
CX Control vs CX 100 µM NMDA	1.00 vs 0.67	2	n.s		
**Figure 1E**	**Mean ± SE**	**n**	**p value**	**ANOVA Table**	**SS**
CX Control vs CX 30 µM NMDA	1.00 ± 0.05 vs 1.03 ± 0.09	12 and 7	n.s	Between columns<br>Within columns<br>Total	0,2666<br>1,771<br>2,038
CX Control vs CX 100 µM NMDA	1.00 ± 0.05 vs 1.20 ± 0.09	12	n.s		
CX 30 µM NMDA vs CX 100 µM NMDA	1.03 ± 0.09 vs 1.20 ± 0.09	7 and 12	n.s		
**Figure 1F**	**Mean ± SE**	**n**	**p value**	**ANOVA Table**	**SS**
HP Control vs HP 30 µM NMDA	1.00 ± 0.06 vs 1.49 ± 0.13	6	n.s	Between columns<br>Within columns<br>Total	2,652<br>1,555<br>4,207
HP Control vs HP 100 µM NMDA	1.00 ± 0.06 vs 1.94 ± 0.18	6	> 0.001		
H HP 30 µM NMDA vs HP 100 µM NMDA	1.49 ± 0.13 vs 1.94 ± 0.18	6	n.s		
**Figure 2A**	**Mean ± SE**	**n**	**p value**	**ANOVA Table**	**SS**
CX Control vs CX 2 µM Ro 106-9920	81.15 ± 1.68 vs 77.87 ± 1.74	5	ns	Between columns<br>Within columns<br>Total	434,1<br>343,9<br>778,0
CX Control vs CX 5 µM Ro 106-9920	81.15 ± 1.68 vs 71.97 ± 2.79	5	ns		
CX Control vs CX 10 µM Ro 106-9920	81.15 ± 1.68 vs 68.70 ± 2.17	5	> 0.01		
HP Control vs HP 2 µM Ro 106-9920	82.52 ± 0.90 vs 76.59 ± 1.46	5	ns	Between columns<br>Within columns<br>Total	451,9<br>171,9<br>623,8
HP Control vs HP 5 µM Ro 106-9920	82.52 ± 0.90 vs 75.63 ± 1.39	5	> 0.05		
HP Control vs HP 10 µM Ro 106-9920	82.52 ± 0.90 vs 69.12 ± 1.93	5	> 0.001		
**Figure 2B**	**Mean ± SE**	**n**	**p value**	**ANOVA Table**	**SS**

CX Control vs CX 30 µM NMDA	74.26 ± 2.47 vs 75.99 ± 2.53	4	n.s	Between columns	143,1
CX Control vs CX Ro 106-9920 + 30 µM NMDA	74.26 ± 2.47 vs 67,96 ± 3.60	4	n.s	Within columns	305,8
CX 30 µM NMDA vs CX Ro 106-9920 + 30 µM NMDA	75.99 ± 2.53 vs 67,96 ± 3.60	4	n.s	Total	448,9
HP Control vs HP 30 µM NMDA	75.54 ± 1.07 vs 66.53 ± 3.12	4	n.s	Between columns	162,8
HP Control vs HP Ro 106-9920 + 30 µM NMDA	75.54 ± 1.07 vs 71,54 ± 3.02	4	n.s	Within columns	239,8
HP 30 µM NMDA vs HP Ro 106-9920 + 30 µM NMDA	66.53 ± 3.12 vs 71,54 ± 3.02	4	n.s	Total	402,6
**Figure 2C**	**Mean ± SE**	**n**	**p value**	**ANOVA Table**	**SS**
CX Control vs CX 100 µM NMDA	75.83 ± 1.52 vs 72.60 ± 3.38	4	n.s	Between columns	776,1
CX Control vs CX Ro 106-9920 + 100 µM NMDA	75.83 ± 1.52 vs 57.39 ± 5.30	4	> 0.05	Within columns	501,3
CX 100 µM NMDA vs CX Ro 106-9920 + 100 µM NMDA	72.60 ± 3.38 vs 57.39 ± 5.30	4	n.s	Total	1277
HP Control vs HP 100 µM NMDA	79.81 ± 0.39 vs 57.54 ± 3.34	4	> 0.01	Between columns	1049
HP Control vs HP Ro 106-9920 + 100 µM NMDA	79.81 ± 0.39 vs 73.30 ± 4.58	4	n.s	Within columns	387,6
HP 100 µM NMDA vs HP Ro 106-9920 + 100 µM NMDA	57.54 ± 3.34 vs 73.30 ± 4.58	4	> 0.05	Total	1437
**Figure 3**	**Mean ± SE**	**n**	**p value**		
CX Control vs CX 30 µM NMDA	1.00 vs 3.16 ± 0.49	3	> 0.01		
HP Control vs HP 30 µM NMDA	1.00 vs 0.20 ± 0.02	3	> 0.001		
CX 30 µM NMDA vs HP 30 µM NMDA	3.16 ± 0.49 vs 0.20 ± 0.02	3	> 0.001		
**Figure 4B**	**Mean ± SE**	**n**	**p value**	**ANOVA Table**	**SS**
CX control vs CX sheNOS	0.01 ± 0.0011 vs 0.0046 ± 0.0014	7 and 5	> 0.05	Between columns	0,0001098
CX shSC vs CX sheNOS	0.0107 ± 0.0004 vs 0.0046 ± 0.0014	4 and 5	> 0.05	Within columns	0,00008698
CX control vs CX shSC	0.01 ± 0.0011 vs 0.0107 ± 0.0004	7 and 4	n.s	Total	0,0001968
**Figure 4D**	**Mean ± SE**	**n**	**p value**		
CX shSC vs CX sheNOS (p65)	1.00 ± 0.04 vs 0.39 ± 0.14	6	> 0.01		
HP shSC vs HP sheNOS (p65)	1.00 ± 0.04 vs 0.42 ± 0.21	4	> 0.05		
CX shSC vs CX sheNOS (Tubulin 1A)	1.00 ± 0.03 vs 0.27 ± 0.17	4	> 0.01		
HP shSC vs HP sheNOS (Tubulin 1A)	1.00 ± 0.07 vs 0.22 ±0.09	4	> 0.001		
**Figure 5A**	**Mean ± SE**	**n**	**p value**	**ANOVA Table**	**SS**
CX Control vs CX LNIO	1.00 ± 0.09 vs 1.12 ± 0.12	6	n.s	Between columns	0,1011
CX Control vs CX 100 µM NMDA	1.00 ± 0.09 vs 1.11 ± 0.14	6	n.s	Within columns	2,267

CX control vs CX LNIO + 100 µM NMDA	1.00 ± 0.09 vs 1.18 ± 0.18	6	n.s	Total	2,369
CX LNIO vs CX 100 µM NMDA	1.12 ± 0.12 vs 1.11 ± 0.14	6	n.s		
CX LNIO vs CX LNIO + 100 µM NMDA	1.12 ± 0.12 vs 1.18 ± 0.18	6	n.s		
CX 100 µM NMDA vs CX LNIO + 100 µM NMDA	1.11 ± 0.14 vs 1.18 ± 0.18	6	n.s		
**Figure 5B**	**Mean ± SE**	**n**	**p value**	**ANOVA Table**	**SS**
HP Control vs HP LNIO	1.00 ± 0.05 vs 1.21 ± 0.28	5	n.s	Between columns<br>Within columns<br>Total	4,148<br>2,694<br>6,842
HP Control vs HP 100 µM NMDA	1.00 ± 0.05 vs 2.06 ± 0.17	5	> 0.01		
HP control vs HP LNIO + 100 µM NMDA	1.00 ± 0.04 vs 1.93 ± 0.17	5	> 0.05		
HP LNIO vs HP 100 µM NMDA	1.21 ± 0.28 vs 2.06 ± 0.17	5	> 0.05		
HP LNIO vs HP LNIO + 100 µM NMDA	1.21 ± 0.28 vs 1.93 ± 0.17	5	n.s		
HP 100 µM NMDA vs HP LNIO + 100 µM NMDA	1.93 ± 0.17 vs 2.06 ± 0.17	5	n.s		
**Figure 5C**	**Mean ± SE**	**n**	**p value**	**ANOVA Table**	**SS**
CX Control vs CX LNIO	1.00 ± 0.05 vs 0.98 ± 0.13	10 and 5	n.s	Between columns<br>Within columns<br>Total	1,572<br>2,104<br>3,675
CX Control vs CX LNIO + 30 µM NMDA	1.00 ± 0.05 vs 1.24 ± 0.11	10 and 5	n.s		
CX Control vs CX LNIO + 100 µM NMDA	1.00 ± 0.05 vs 1.52 ± 0.13	10 and 9	> 0.01		
CX LNIO vs CX LNIO + 30 µM NMDA	0.98 ± 0.13 vs 1.24 ± 0.11	5	n.s		
CX LNIO vs CX LNIO + 100 µM NMDA	0.98 ± 0.13 vs 1.52 ± 0.13	5 and 9	> 0.01		
CX LNIO + 30 µM NMDA vs CX LNIO + 100 µM NMDA	1.24 ± 0.11 vs 1.52 ± 0.13	5 and 9	n.s		
**Figure 5D**	**Mean ± SE**	**n**	**p value**	**ANOVA Table**	**SS**
HP Control vs HP LNIO	1.00 ± 0.04 vs 1.04 ± 0.16	6 and 4	n.s	Between columns<br>Within columns<br>Total	1,067<br>0,4511<br>1,518
HP Control vs HP LNIO + 30 µM NMDA	1.00 ± 0.04 vs 1.27 ± 0.06	6 and 4	n.s		
HP Control vs HP 100 µM NMDA	1.00 ± 0.04 vs 1.62 ± 0.07	6 and 4	> 0.01		
HP LNIO vs HP LNIO 30 µM NMDA	1.04 ± 0.16 vs 1.27 ± 0.06	4	n.s		
HP LNIO vs HP LNIO 100 µM NMDA	1.04 ± 0.16 vs 1.62 ± 0.07	4	> 0.01		
HP LNIO + 30 µM NMDA vs HP LNIO + 100 µM NMDA	1.27 ± 0.06 vs 1.62 ± 0.07	4	n.s		
**Figure 5E**	**Mean ± SE**	**n**	**p value**	**ANOVA Table**	**SS**
HP Control vs HP SNAP	1.00 ± 0.06 vs 1.07 ± 0.11	6 and 5	n.s	Between columns<br>Within columns<br>Total	2,107<br>1,857<br>3,964
HP Control vs HP 100 µM NMDA	1.00 ± 0.06 vs 1.76 ± 0.17	6	> 0.01		
HP Control vs HP SNAP + 100 µM NMDA	1.00 ± 0.06 vs 1.20 ± 0.14	6	n.s		

HP SNAP vs HP 100 µM NMDA	1.07 ± 0.11 vs 1.76 ± 0.17	5 and 6	> 0.01		
HP SNAP vs HP SNAP + 100 µM NMDA	1.07 ± 0.11 vs 1.20 ± 0.14	5 and 6	n.s		
HP 100 µM NMDA vs HP SNAP + 100 µM NMDA	1.76 ± 0.17 vs 1.20 ± 0.14	6	> 0.05		
**Figure 5F**	**Mean ± SE**	**n**	**p value**	**ANOVA Table**	**SS**
HP Control vs HP 100 µM NMDA	1.00 vs 2.32 ± 0.31	8	> 0.001	Between columns<br>Within columns<br>Total	SS<br>11,95<br>5,726<br>17,68
HP Control vs HP Ro 106-9920	1.00 vs 0.55 ± 0.12	8 and 4	n.s		
HP Control vs HP Ro106-9920 + 100 µM NMDA	1.00 vs 0.88 ± 0.16	8 and 4	n.s		
HP 100 µM NMDA vs HP Ro 106-9920	2.32 ± 0.31 vs 0.55 ± 0.12	8 and 4	> 0.001		
HP 100 µM NMDA vs HP Ro106-9920 + 100 µM NMDA	2.32 ± 0.31 vs 0.88 ± 0.16	8 and 4	> 0.001		
HP Ro106-9920 vs HP Ro106-9920 + 100 µM NMDA	0.55 ± 0.12 vs 0.88 ± 0.16	4	n.s		
**Figure 5G**	**Mean ± SE**	**n**	**p value**	**ANOVA Table**	**SS**
HP Control vs HP 100 µM NMDA	1.00 vs 2.32 ± 0.31	8	> 0.001	Between columns<br>Within columns<br>Total	12,62<br>8,880<br>21,50
HP Control vs HP SNAP	1.00 vs 0.76 ± 0.13	8 and 4	n.s		
HP Control vs HP SNAP + 100 µM NMDA	1.00 vs 1.10 ± 0.40	8 and 5	n.s		
HP 100 µM NMDA vs HP SNAP	2.48 ± 0.31 vs 0.76 ± 0.13	8 and 4	> 0.01		
HP 100 µM NMDA vs HP SNAP + 100 µM NMDA	2.48 ± 0.31 vs 1.10 ± 0.40	8 and 5	> 0.01		
HP SNAP vs HP SNAP + 100 µM NMDA	0.76 ± 0.13 vs 1.10 ± 0.40	4 and 5	n.s		
**Figure 6D**	**Mean ± SE**	**n**	**p value**		
CX Control vs CX 30 µM NMDA (GluN2A)	1.00 vs 1.86 ± 0.33	5	> 0.05		
HP Control vs HP 30 µM NMDA (GluN2A)	1.00 vs 1.76 ± 0.15	5	> 0.01		
CX 30 µM NMDA vs HP 30 µM NMDA (GluN2A)	1.86 ± 0.33 vs 1.76 ± 0.15	5	n.s		
CX Control vs CX 30 µM NMDA (PSD95)	1.00 vs 2.02 ± 0.01	3	> 0.001		
HP Control vs HP 30 µM NMDA (PSD95)	1.00 vs 1.70 ± 0.13	3	> 0.01		
CX 30 µM NMDA vs HP 30 µM NMDA (PSD95)	2.02 ± 0.01 vs 1.70 ± 0.13	3	n.s		
CX Control vs CX 30 µM NMDA (SAPAP4)	1.00 vs 1.58 ± 0.09	4	> 0.001		
HP Control vs HP 30 µM NMDA (SAPAP4)	1.00 vs 1.15 ± 0.06	3	n.s		
CX 30 µM NMDA vs HP 30 µM NMDA (SAPAP4)	1.58 ± 0.09 vs 1.15 ± 0.06	3 and 4	> 0.05		
**Figure S1D**	**Mean ± SE**	**n**	**p value**	**ANOVA Table**	**SS**
CX Control vs CX 30 µM NMDA	72.58 ± 2.61 vs 68.94 ± 1.23	3	n.s	Between columns	185,1

CX Control vs CX 60 µM NMDA	72.58 ± 2.61 vs 63.26 ± 3.75	3	n.s	Within columns<br>Total	911,3<br>1096
CX Control vs CX 100 µM NMDA	72.58 ± 2.61 vs 65.04 ± 4.66	3	n.s		
CX 30 µM NMDA vs CX 60 µM NMDA	68.94 ± 1.23 vs 63.26 ± 3.75	3	n.s		
CX 30 µM NMDA vs CX 100 µM NMDA	68.94 ± 1.23 vs 65.04 ± 4.66	3	n.s		
CX 60 µM NMDA vs CX 100 µM NMDA	63.26 ± 3.75 vs 65.04 ± 4.66	3	n.s		
HP Control vs HP 30 µM NMDA	73.90 ± 3.96 vs 70.61 ± 4.14	4	n.s	Between columns<br>Within columns<br>Total	1049<br>387,6<br>1437
HP Control vs HP 60 µM NMDA	73.90 ± 3.96 vs 53.25 ± 5.15	4	> 0.05		
HP Control vs HP 100 µM NMDA	73.90 ± 3.96 vs 40.12 ± 4.53	4	> 0.01		
HP 30 µM NMDA vs HP 60 µM NMDA	70.61 ± 4.14 vs 53.25 ± 5.15	4	n.s		
HP 30 µM NMDA vs HP 100 µM NMDA	70.61 ± 4.14 vs 40.12 ± 4.53	4	> 0.01		
HP 60 µM NMDA vs HP 100 µM NMDA	53.25 ± 5.15 vs 40.12 ± 4.53	4	n.s		
**Figure S1E**	**Mean ± SE**	**n**	**p value**		
Control vs NMDA	1.00 ± 0.13 vs 18.62 ± 2.79	13	> 0.001		
**Figure S2C**	**Mean ± SE**	**n**	**p value**		
HP Control vs HP NMDA (Neurons)	1.00 vs 1.75 ± 0.26	3	> 0.05		
HP Control vs HP NMDA (Astrocytes)	1.00 vs 1.48 ± 0.17	3	> 0.05		
**Figure S3A**	**Mean ± SE**	**n**	**p value**	**ANOVA Table**	**SS**
CX Control vs CX LNIO	1.00 ± 0.09 vs 1.10 ± 0.13	5	n.s	Between columns<br>Within columns<br>Total	0,3149<br>1,309<br>1,624
CX Control vs CX 100 µM NMDA	1.00 ± 0.09 vs 1.08 ± 0.15	5	n.s		
CX Control vs CX LNIO + 100 µM NMDA	1.00 ± 0.09 vs 0.78 ± 0.14	5	n.s		
CX LNIO vs CX 100 µM NMDA	1.10 ± 0.13 vs 1.08 ± 0.15	5	n.s		
CX LNIO vs CX LNIO + 100 µM NMDA	1.10 ± 0.13 vs 0.78 ± 0.14	5	n.s		
CX 100 µM NMDA vs CX LNIO + 100 µM NMDA	1.08 ± 0.15 vs 0.78 ± 0.14	5	n.s		
**Figure S3B**	**Mean ± SE**	**n**	**p value**	**ANOVA Table**	**SS**
HP Control vs HP LNIO	1.00 ± 0.08 vs 0.94 ± 0.15	4	n.s	Between columns<br>Within columns<br>Total	0,009124<br>0,7017<br>0,7108
HP Control vs HP 100 µM NMDA	1.00 ± 0.08 vs 0.96 ± 0.14	4	n.s		
HP Control vs HP LNIO + 100 µM NMDA	1.00 ± 0.08 vs 0.94 ± 0.09	4	n.s		
HP LNIO vs HP 100 µM NMDA	0.94 ± 0.15 vs 0.96 ± 0.14	4	n.s		
HP LNIO vs HP LNIO + 100 µM NMDA	0.94 ± 0.15 vs 0.94 ± 0.09	4	n.s		

HP 100 µM NMDA vs HP LNIO + 100 µM NMDA	0.96 ± 0.14 vs 0.94 ± 0.09	4	n.s		
**Figure S3C**	**Mean ± SE**	**n**	**p value**	**ANOVA Table**	**SS**
CX Control vs CX LNIO	1.00 ± 0.19 vs 0.71 ± 0.12	6	n.s	Between columns	0,5933
CX Control vs CX 100 µM NMDA	1.00 ± 0.19 vs 0.88 ± 0.19	6	n.s	Within columns	2,799
CX Control vs CX LNIO + 100 µM NMDA	1.00 ± 0.19 vs 0.59 ± 0.08	6	n.s	Total	3,392
CX LNIO vs CX 100 µM NMDA	0.71 ± 0.12 vs 0.88 ± 0.19	6	n.s		
CX LNIO vs CX LNIO + 100 µM NMDA	0.71 ± 0.12 vs 0.59 ± 0.08	6	n.s		
CX 100 µM NMDA vs CX LNIO + 100 µM NMDA	0.88 ± 0.19 vs 0.59 ± 0.08	6	n.s		
**Figure S3D**	**Mean ± SE**	**n**	**p value**	**ANOVA Table**	**SS**
HP Control vs HP LNIO	1.00 ± 0.19 vs 0.98 ± 0.20	5	n.s	Between columns	1,445
HP Control vs HP 100 µM NMDA	1.00 ± 0.19 vs 1.64 ± 0.51	5	n.s	Within columns	7,858
HP Control vs HP LNIO + 100 µM NMDA	1.00 ± 0.19 vs 1.10 ± 0.23	5	n.s	Total	9,303
HP LNIO vs HP 100 µM NMDA	0.98 ± 0.19 vs 1.64 ± 0.51	5	n.s		
HP LNIO vs HP LNIO + 100 µM NMDA	0.98 ± 0.19 vs 1.10 ± 0.23	5	n.s		
HP 100 µM NMDA vs HP LNIO + 100 µM NMDA	1.64 ± 0.51 vs 1.10 ± 0.23	5	n.s		
**Figure S4A**	**Mean ± SE**	**n**	**p value**	**ANOVA Table**	**SS**
HP Control vs 100 µM NMDA 1h	1.00 vs 1.25 ± 0.31	4	n.s	Between columns	5,784
HP Control vs 100 µM NMDA 2h	1.00 vs 2.58 ± 0.38	4	> 0.01	Within columns	3,253
HP Control vs 100 µM NMDA 6h	1.00 vs 1.64 ± 0.26	4	n.s	Total	9,037
**Figure S4B**	**Mean ± SE**	**n**	**p value**	**ANOVA Table**	**SS**
HP Control vs 100 µM NMDA 1h	1.00 vs 1.00 ± 0.47	2	n.s	Between columns	0,01717
HP Control vs 100 µM NMDA 2h	1.00 vs 0.91 ± 0.02	3	n.s	Within columns	0,4623
HP Control vs 100 µM NMDA 6h	1.00 vs 0.99 ± 0.04	3	n.s	Total	0,4795

Supplemental Table 1. Statistics of all experiments

SUPPLEMENTAL TABLE 2

		Treatment			
		Hippocampal Cultures		Cortical Cultures	
**Protein name**	**Entry name**	**HP Control**	**HP NMDA**	**CX Control**	**CX NMDA**
**n**		6	5	6	6
14-3-3 protein epsilon	1433E_RAT	5	3	2	3
14-3-3 protein eta	1433F_RAT	5	3	2	2
14-3-3 protein gamma	1433G_RAT	6	4	2	2
14-3-3 protein theta	1433T_RAT	6	4	2	2
14-3-3 protein zeta/delta	1433Z_RAT	0	0	6	6
1-phosphatidylinositol-4,5-bisphosphate phosphodiesterase beta-1	PLCB1_RAT	0	1	0	0
26S protease regulatory subunit 4	PRS4_RAT	1	1	0	0
26S protease regulatory subunit 6A	PRS6A_RAT	0	0	2	2
26S protease regulatory subunit 6B	PRS6B_RAT	0	0	3	3
26S protease regulatory subunit 7	PRS7_RAT	1	2	0	0
26S protease regulatory subunit 8	PRS8_RAT	0	0	1	2
26S proteasome non-ATPase regulatory subunit 11	PSD11_RAT	0	0	1	1
26S proteasome non-ATPase regulatory subunit 13	PSD13_RAT	0	0	0	1
26S proteasome non-ATPase regulatory subunit 2	PSMD2_RAT	1	3	2	3
2-oxoglutarate dehydrogenase, mitochondrial	ODO1_RAT	0	0	1	2

40S ribosomal protein S17	RS17_RAT	1	0	0	0
40S ribosomal protein S3	RS3_RAT	0	0	2	3
40S ribosomal protein S4, X isoform	RS4X_RAT	0	0	0	2
40S ribosomal protein S8	RS8_RAT	0	0	1	0
40S ribosomal protein SA	RSSA_RAT	3	2	3	3
4-aminobutyrate aminotransferase, mitochondrial	GABT_RAT	0	0	2	3
4F2 cell-surface antigen heavy chain	4F2_RAT	4	4	2	3
4-trimethylaminobutyraldehyde dehydrogenase	AL9A1_RAT	0	0	1	2
60 kDa heat shock protein, mitochondrial	CH60_RAT	4	4	3	3
60S acidic ribosomal protein P0	RLA0_RAT	0	0	3	3
60S ribosomal protein L19	RL19_RAT	0	0	1	0
60S ribosomal protein L23a	RL23A_RAT	0	1	0	0
60S ribosomal protein L3	RL3_RAT	0	0	3	2
60S ribosomal protein L4	RL4_RAT	0	0	2	2
60S ribosomal protein L5	RL5_RAT	0	0	1	0
6-phosphofructokinase	K6PP_RAT	1	3	0	1
6-phosphofructokinase, liver type	PFKAL_RAT	4	3	1	2
6-phosphofructokinase, muscle type	PFKAM_RAT	0	0	2	2
6-phosphogluconate dehydrogenase, decarboxylating	6PGD_RAT	4	2	3	2
6-phosphogluconolactonase	6PGL_RAT	0	0	0	1
78 kDa glucose-regulated protein	GRP78_RAT	0	0	1	3

7-dehydrocholesterol reductase	DHCR7_RAT	2	0	1	0
Acetyl-CoA acetyltransferase, cytosolic	THIC_RAT	0	0	2	3
Acetyl-CoA acetyltransferase, mitochondrial	THIL_RAT	0	0	1	3
Aconitate hydratase, mitochondrial	ACON_RAT	5	2	3	3
Actin cytoplasmic 2	ACTG_RAT	0	0	1	2
Actin, alpha cardiac muscle 1	ACTC_RAT	0	0	0	2
Actin, alpha skeletal muscle	ACTS_RAT	0	1	0	0
Actin-related protein 2	ARP2_RAT	0	0	2	2
Actin-related protein 2/3 complex subunit 1A	ARC1A_RAT	0	0	1	3
Adenosylhomocysteinase	SAHH_RAT	4	2	1	0
Adenylate kinase isoenzyme 1	KAD1_RAT	0	1	0	0
Adenylyl cyclase-associated protein 1	CAP1_RAT	2	3	3	3
ADP/ATP translocase 1	ADT1_RAT	0	0	3	3
ADP/ATP translocase 2	ADT2_RAT	2	2	1	1
Alanine--tRNA ligase, cytoplasmic	SYAC_RAT	0	0	3	3
Alcohol dehydrogenase [NADP(+)]	ADHX_RAT	0	0	1	2
Aldehyde dehydrogenase, mitochondrial	ALDH2_RAT	2	2	1	3
Alpha-actinin-4	ACTN4_RAT	0	0	1	2
Alpha-aminoadipic semialdehyde dehydrogenase	AL7A1_RAT	1	1	1	0
Alpha-centractin	ACTZ_RAT	0	0	3	3
Alpha-tubulin N-acetyltransferase 1	ATAT_RAT	0	0	2	0

Amine oxidase [flavin-containing] B	AOFB_RAT	4	2	0	0
Amphiphysin	AMPH_RAT	0	1	0	0
Anionic trypsin-1	TRY1_RAT	0	0	0	1
AP-2 complex subunit alpha-2	AP2A2_RAT	0	0	3	3
AP-2 complex subunit beta	AP2B1_RAT	0	0	3	3
AP2-associated protein kinase 1	AAK1_RAT	0	0	1	1
ARF GTPase-activating protein GIT1	APOE_RAT	0	0	1	1
ArfGAP with GTPase domain, ankyrin repeat and PH domain 3	ARGI1_RAT	0	0	1	0
Arginine--tRNA ligase, cytoplasmic	SYRC_RAT	0	0	1	0
Armadillo repeat-containing protein 10	ARM10_RAT	0	0	0	1
Asparaginyl-tRNA synthetase	AATC_RAT	0	0	0	3
Aspartate aminotransferase, mitochondrial	AATM_RAT	4	4	3	3
Aspartate--tRNA ligase, cytoplasmic	SYDC_RAT	0	0	1	0
ATP synthase F(0) complex subunit B1, mitochondrial	AT5F1_RAT	0	0	1	1
ATP synthase subunit gamma, mitochondrial	ATPG_RAT	3	4	1	2
Beta-soluble NSF attachment protein	SNAB_RAT	0	0	0	1
Bleomycin hydrolase	BLMH_RAT	0	0	1	1
Brain acid soluble protein 1	BASP1_RAT	0	2	0	0
Calcium/calmodulin-dependent protein kinase type II subunit alpha	KCC2A_RAT	0	0	3	3
Calnexin	CALX_RAT	0	0	2	3

Calpain-2 catalytic subunit	CAN2_RAT	0	0	0	2
Calreticulin	CALR_RAT	0	0	2	3
CaM kinase-like vesicle-associated protein	CAMKV_RAT	0	0	0	1
cAMP-dependent protein kinase catalytic subunit beta	KAPCB_RAT	0	0	2	3
Casein kinase II subunit alpha	CSK21_RAT	0	0	0	2
Catalase	CATA_RAT	0	0	1	1
Catenin beta-1	CTNB1_RAT	0	0	2	3
Cathepsin D	CATD_RAT	4	1	2	2
Cell adhesion molecule 3	CADM3_RAT	0	0	1	1
cGMP-dependent 3',5'-cyclic phosphodiesterase	PDE2A_RAT	0	0	1	2
Citrate synthase, mitochondrial	CISY_RAT	4	3	0	0
Clathrin coat assembly protein AP180	AP180_RAT	1	3	0	0
CLIP-associating protein 2	CLAP2_RAT	0	0	2	3
Coatomer subunit beta	COPB_RAT	0	0	1	0
Coatomer subunit gamma-1	COPG1_RAT	0	0	2	3
Contactin-1	CNTN1_RAT	0	0	3	3
Coronin-1A	COR1A_RAT	3	2	3	3
Creatine kinase M-type	KCRM_RAT	0	1	0	0
Cullin-3	CUL3_RAT	0	0	1	0
Cullin-associated NEDD8-dissociated protein 1	CAND1_RAT	0	0	2	3
Cytochrome b-c1 complex subunit 1, mitochondrial	QCR1_RAT	0	0	3	3

Cytochrome b-c1 complex subunit 2, mitochondrial	QCR2_RAT	2	4	2	3
Cytosolic non-specific dipeptidase	CNDP2_RAT	0	0	2	0
D-3-phosphoglycerate dehydrogenase	SERA_RAT	3	4	3	3
D-beta-hydroxybutyrate dehydrogenase, mitochondrial	BDH_RAT	0	1	0	0
Dihydrolipoyl dehydrogenase, mitochondrial	DLDH_RAT	0	0	1	1
Dihydropyrimidinase-related protein 1	DPYL1_RAT	0	0	3	3
Dihydropyrimidinase-related protein 4 (Fragment)	DPYL4_RAT	3	3	2	1
Dihydropyrimidinase-related protein 5	DPYL5_RAT	5	4	1	1
Dipeptidyl peptidase 2	DPP2_RAT	3	2	1	0
Dipeptidyl peptidase 3	DPP3_RAT	0	0	1	3
Discs, large (Drosophila) homolog-associated protein 4	DLGP4_RAT	0	0	0	4
DnaJ homolog subfamily A member 1	DNJA1_RAT	2	3	0	0
DnaJ homolog subfamily A member 2	DNJA2_RAT	0	0	1	3
Dolichyl-diphosphooligosaccharide--protein glycosyltransferase subunit 1	RPN1_RAT	1	3	0	0
Dynactin subunit 1	DCTN1_RAT	0	0	0	2
Dynamin-1	DYN1_RAT	4	3	0	0
Dynamin-like 120 kDa protein, mitochondrial	OPA1_RAT	0	0	2	3
EH domain-containing protein 1	EHD1_RAT	0	0	1	2
EH domain-containing protein 3	EHD3_RAT	1	0	1	1
Electron transfer flavoprotein subunit alpha, mitochondrial	ETFA_RAT	3	2	0	0
Elongation factor 1-alpha 2	EF1A2_RAT	0	1	0	1

Elongation factor 1-delta	EF1D_RAT	3	2	2	3
Elongation factor 1-gamma	EF1G_RAT	0	0	3	3
Elongation factor 2	EF2_RAT	0	0	3	3
Elongation factor Tu, mitochondrial	EFTU_RAT	0	0	2	3
Endoplasmin	ENPL_RAT	0	0	2	2
Epsin-1	EPN1_RAT	0	0	1	0
ES1 protein homolog, mitochondrial	ES1_RAT	0	0	0	1
Eukaryotic initiation factor 4A-II	IF4A2_RAT	0	0	3	3
Eukaryotic translation initiation factor 2 subunit 3	IF2G_RAT	0	0	2	1
Eukaryotic translation initiation factor 3 subunit A	EIF3A_RAT	0	0	1	2
Eukaryotic translation initiation factor 3 subunit B	EIF3B_RAT	0	1	0	1
Eukaryotic translation initiation factor 5B	IF2P_RAT	0	1	0	0
Excitatory amino acid transporter 1	EAA1_RAT	3	3	0	0
Exocyst complex component 4	EXOC4_RAT	0	0	1	0
Exportin-1	EXPO1_RAT	0	0	1	3
Ezrin	EZRI_RAT	0	0	1	0
F-actin-capping protein subunit alpha-2	CAZA2_RAT	0	0	2	2
F-actin-capping protein subunit beta	CAPZB_RAT	0	0	0	1
Fascin	FSCN1_RAT	5	3	3	3
Fatty acid synthase	FAS_RAT	1	3	3	3
Fatty acid-binding protein, brain	FABP7_RAT	1	0	0	0

FERM, RhoGEF and pleckstrin domain-containing protein 1	FARP1_RAT	0	0	0	1
Fructose-bisphosphate aldolase A	ALDOA_RAT	0	0	3	3
Fructose-bisphosphate aldolase C	ALDOC_RAT	0	0	3	3
Fumarate hydratase, mitochondrial	FUMH_RAT	2	1	0	0
Gamma-enolase	ENOG_RAT	4	4	3	3
General vesicular transport factor p115	USO1_RAT	0	0	1	1
Glucose-6-phosphate 1-dehydrogenase	G6PD_RAT	0	0	0	1
Glucose-6-phosphate isomerase	G6PI_RAT	4	4	2	3
Glutamate decarboxylase 2	DCE2_RAT	0	0	1	0
Glutamate dehydrogenase 1, mitochondrial	DHE3_RAT	4	4	3	3
Glutaminase kidney isoform, mitochondrial	GLSK_RAT	1	2	0	1
Glutamine synthetase	GLNA_RAT	3	4	0	2
Glutathione S-transferase alpha-3	GSTA3_RAT	1	2	0	0
Glutathione S-transferase Mu 1	GSTM1_RAT	2	2	0	0
Glutathione S-transferase Mu 5	GSTM5_RAT	0	2	0	0
Glutathione S-transferase P	GSTP1_RAT	0	0	1	1
Glutathione S-transferase Yb-3	GSTM4_RAT	0	0	0	1
Glyceraldehyde-3-phosphate dehydrogenase	G3P_RAT	0	0	2	3
Glycine amidinotransferase, mitochondrial	GATM_RAT	0	0	2	3
Glycine--tRNA ligase	GARS_RAT	0	0	1	2
Glycogen phosphorylase, brain form (Fragment)	PYGB_RAT	0	2	1	3

Glycogen synthase kinase-3 beta	GSK3B_RAT	0	0	2	3
GPI inositol-deacylase	PGAP1_RAT	0	0	1	0
GTP-binding nuclear protein Ran	RAN_RAT	0	0	2	1
Guanine deaminase	GUAD_RAT	1	1	0	0
Guanine nucleotide-binding protein G(i) subunit alpha-1	GNAI1_RAT	0	0	3	3
Guanine nucleotide-binding protein G(i) subunit alpha-2	GNAI2_RAT	0	0	2	2
Guanine nucleotide-binding protein G(I)/G(S)/G(T) subunit beta-1	GBB1_RAT	4	4	3	3
Guanine nucleotide-binding protein G(I)/G(S)/G(T) subunit beta-2	GBB2_RAT	3	3	2	3
Guanine nucleotide-binding protein G(o) subunit alpha	GNAO_RAT	0	0	2	3
Guanine nucleotide-binding protein G(s) subunit alpha isoforms short	GNAS2_RAT	0	0	2	3
Guanine nucleotide-binding protein G(z) subunit alpha	GNAZ_RAT	0	0	0	1
Guanine nucleotide-binding protein subunit alpha-12	GNA12_RAT	0	0	1	0
Guanine nucleotide-binding protein subunit beta-2-like 1	GBLP_RAT	3	3	1	2
Guanine nucleotide-binding protein-like 1	GNL1_RAT	0	0	1	0
Guanylate cyclase soluble subunit alpha-3	GCYA3_RAT	0	0	1	0
Heat shock 70 kDa protein 4	HSP74_RAT	4	2	0	0
Heat shock cognate 71 kDa	HSP7C_RAT	0	0	3	3
Heat shock protein 105 kDa	HS105_RAT	0	1	0	0
Heat shock protein 75 kDa, mitochondrial	TRAP1_RAT	0	0	1	2

Heat shock protein HSP 90-alpha	HS90A_RAT	0	0	3	3
Heat shock protein HSP 90-beta	HS90B_RAT	0	0	3	3
Heterogeneous nuclear ribonucleoprotein A1	ROA1_RAT	0	2	0	0
Heterogeneous nuclear ribonucleoprotein A3	ROA3_RAT	0	0	0	1
Heterogeneous nuclear ribonucleoprotein K	HNRPK_RAT	0	0	2	3
Hexokinase-1	HXK1_RAT	5	4	3	3
High mobility group protein B1	HMGB1_RAT	0	2	0	0
Histone H1.4	H14_RAT	0	0	1	0
Histone H4	H4_RAT	0	0	1	0
Hsc70-interacting protein	F10A1_RAT	1	0	0	0
Hsp90 co-chaperone Cdc37	CDC37_RAT	0	0	0	1
Hydroxymethylglutaryl-CoA synthase, cytoplasmic	HMCS1_RAT	2	3	3	3
Hypoxanthine-guanine phosphoribosyltransferase	HPRT_RAT	0	1	0	0
Ig kappa chain C region, A allele	KACA_RAT	0	1	0	0
Importin subunit beta-1	IMB1_RAT	0	0	2	3
Inosine triphosphate pyrophosphatase	ITPA_RAT	0	0	0	1
Insulin receptor-related protein	INSRR_RAT	0	1	0	0
Isocitrate dehydrogenase [NAD] subunit alpha, mitochondrial	IDH3A_RAT	0	1	0	0
Isocitrate dehydrogenase [NAD] subunit gamma 1, mitochondrial	IDHG1_RAT	0	0	1	0
Isocitrate dehydrogenase [NADP] cytoplasmic	IDHC_RAT	3	3	0	2

Isocitrate dehydrogenase [NADP], mitochondrial	IDHP_RAT	2	4	0	0
Isoform 1 of Cytosolic acyl coenzyme A thioester hydrolase	BACH_RAT	0	0	2	3
Isoform 11a of Neurexin-1	NRX1A_RAT	0	0	2	2
Isoform 13 of Dynamin-3	DYN3_RAT	0	0	0	2
Isoform 2 of Bifunctional protein NCOAT	NCOAT_RAT	0	0	1	1
Isoform 2 of Clathrin coat assembly protein AP180	AP180_RAT	0	0	2	2
Isoform 2 of Dynamin-1-like protein	DNM1L_RAT	0	0	2	0
Isoform 2 of Endoplasmin	ENPL_RAT	0	0	1	1
Isoform 2 of Glial fibrillary acidic protein	GFAP_RAT	0	0	2	5
Isoform 2 of Glutaredoxin-3	GLRX3_RAT	0	0	0	1
Isoform 2 of Heterogeneous nuclear ribonucleoprotein A3	ROA3_RAT	0	0	0	1
Isoform 2 of Microtubule-associated protein 6	MAP6_RAT	0	0	3	3
Isoform 2 of Neuronal membrane glycoprotein M6-a	GPM6A_RAT	1	2	1	0
Isoform 2 of Phytanoyl-CoA hydroxylase-interacting protein-like	PHIPL_RAT	0	0	1	0
Isoform 2 of Protein transport protein Sec31A	SC31A_RAT	0	0	0	1
Isoform 2 of Reticulon-3	RTN3_RAT	0	0	1	1
Isoform 2 of Sarcoplasmic/endoplasmic reticulum calcium ATPase 2	AT2A2_RAT	0	0	1	3
Isoform 2 of Serine/threonine-protein phosphatase 2B catalytic subunit alpha isoform	PP2BA_RAT	0	0	1	0
Isoform 2 of SRC kinase signaling inhibitor 1	SRCN1_RAT	0	0	1	0

Isoform 2 of Voltage-dependent anion-selective channel protein 3	VDAC3_RAT	0	1	0	0
Isoform 2 of WD repeat-containing protein 7	WDR7_RAT	0	0	1	1
Isoform 3 of Dynamin-1	DYN1_RAT	0	0	2	1
Isoform 3 of NADH-cytochrome b5 reductase 3	NB5R3_RAT	0	0	1	3
Isoform 3 of Neuronal-specific septin-3	SEPT3_RAT	0	0	2	1
Isoform 3 of Septin-11	SEP11_RAT	0	0	3	3
Isoform 4 of Dynamin-1-like protein	DNM1L_RAT	0	0	0	2
Isoform 4 of Heterogeneous nuclear ribonucleoprotein D0	HNRPD_RAT	0	0	1	0
Isoform 4 of Protein NDRG4	NDRG4_RAT	0	0	0	1
Isoform 5 of Disks large homolog 2	DLG2_RAT	0	0	0	1
Isoform 5 of Dynamin-1-like protein	DNM1L_RAT	0	0	1	1
Isoform 5 of Synaptojanin-1	SYNJ1_RAT	0	0	1	2
Isoform Cytoplasmic of Fumarate hydratase, mitochondrial	FUMH_RAT	0	0	2	2
Isoform Delta 5 of Calcium/calmodulin-dependent protein kinase type II subunit delta	KCC2D_RAT	0	0	1	3
Isoform E1 of Drebrin	DREB_RAT	0	0	1	0
Isoform GLAST-1A of Excitatory amino acid transporter 1	EAA1_RAT	0	0	2	3
Isoform IB of Synapsin-1	SYN1_RAT	0	0	3	3
Isoform II of V-type proton ATPase 116 kDa subunit a isoform 1	VPP1_RAT	0	0	3	3
Isoform IIb of Synapsin-2	SYN2_RAT	0	0	2	3

Isoform M2 of Pyruvate kinase isozymes M1/M2	KPYM_RAT	3	3	0	0
Isoform Short of 14-3-3 protein beta/alpha	1433B_RAT	0	0	2	2
Isoform ZA of Plasma membrane calcium-transporting ATPase 2	AT2B2_RAT	0	0	2	0
Isoform ZA of Plasma membrane calcium-transporting ATPase 4	AT2B4_RAT	0	0	3	3
Kinesin-1 heavy chain	KINH_RAT	0	0	0	1
Lactoylglutathione lyase	LGUL_RAT	0	0	1	0
Lamin-B1	LMNB1_RAT	0	1	0	0
Large neutral amino acids transporter small subunit 1	LAT1_RAT	1	0	2	3
Latexin	LXN_RAT	0	0	0	1
LETM1 and EF-hand domain-containing protein 1, mitochondrial	LETM1_RAT	0	0	1	2
Leucine-rich PPR motif-containing protein, mitochondrial	LPPRC_RAT	1	3	0	0
Leucine-rich repeat-containing protein 14 [LRC14_RAT]	LRC14_RAT	0	0	0	2
L-lactate dehydrogenase A chain	LDHA_RAT	0	0	1	3
L-lactate dehydrogenase B chain	LDHB_RAT	5	4	3	3
Lon protease homolog, mitochondrial	LONM_RAT	0	0	1	1
Long-chain specific acyl-CoA dehydrogenase, mitochondrial	ACADL_RAT	3	1	0	0
Long-chain-fatty-acid--CoA ligase 4	ACSL4_RAT	0	0	1	1
Lysosome-associated membrane glycoprotein 1	LAMP1_RAT	2	3	1	1
Malate dehydrogenase, cytoplasmic	MDHC_RAT	5	4	3	3

Malate dehydrogenase, mitochondrial	MDHM_RAT	5	4	3	3
Matrin-3	MATR3_RAT	0	0	1	2
Microtubule-actin cross-linking factor 1	MACF1_RAT	0	0	1	1
Microtubule-associated protein 1A	MAP1A_RAT	2	2	1	2
Microtubule-associated protein 1S	MAP1S_RAT	0	0	1	0
Microtubule-associated protein 4	MAP4_RAT	0	0	3	3
Microtubule-associated protein RP/EB family member 1	MARE1_RAT	0	0	3	2
Microtubule-associated protein tau	TAU_RAT	3	2	0	0
Mitochondrial import receptor subunit TOM70	TOM70_RAT	2	2	0	0
Mitochondrial inner membrane protein (Fragment)	IMMT_RAT	0	1	2	2
Mitogen-activated protein kinase 1	MK01_RAT	0	0	2	3
Mitogen-activated protein kinase 3	MK03_RAT	0	0	1	3
Monocarboxylate transporter 1	MOT1_RAT	0	0	1	1
Monocarboxylate transporter 2	MOT2_RAT	0	0	0	1
Mu-crystallin homolog	CRYM_RAT	3	4	0	0
Multifunctional protein ADE2	PUR6_RAT	0	0	0	2
Myelin transcription factor 1-like protein	MYT1L_RAT	0	0	3	1
Myosin light chain 1/3, skeletal muscle isoform	MYL1_RAT	0	1	0	0
Myosin regulatory light chain 2, skeletal muscle isoform	MLRS_RAT	0	1	0	0
Myosin-3	MYH3_RAT	0	1	0	0
Myosin-4	MYH4_RAT	0	1	0	0

Myosin-9	MYH9_RAT	1	0	0	0
Myristoylated alanine-rich C-kinase substrate	MARCS_RAT	0	2	0	0
NADH dehydrogenase [ubiquinone] 1 alpha subcomplex subunit 9, mitochondrial	NDUA9_RAT	0	0	1	3
NADH dehydrogenase [ubiquinone] iron-sulfur protein 2, mitochondrial	NDUS2_RAT	0	0	0	1
NADH-ubiquinone oxidoreductase 75 kDa subunit, mitochondrial	NDUS1_RAT	0	0	3	3
Neogenin (Fragment)	NEO1_RAT	0	0	0	1
Neural cell adhesion molecule 1	NCAM1_RAT	3	4	0	0
Neurochondrin	NCDN_RAT	3	2	2	3
Neurofilament heavy polypeptide	NFH_RAT	0	0	0	2
Neuromodulin	NEUM_RAT	0	0	3	3
Neuronal membrane glycoprotein M6-a	GPM6A_RAT	0	0	1	3
Non-POU domain-containing octamer-binding protein	NONO_RAT	3	3	0	0
Nucleolin	NUCL_RAT	0	0	2	2
Nucleoside diphosphate kinase B	NDKB_RAT	0	1	0	0
Nucleosome assembly protein 1-like 4	NP1L4_RAT	0	0	0	1
Obg-like ATPase 1	OLA1_RAT	0	0	0	3
Ornithine aminotransferase, mitochondrial	OAT_RAT	0	0	1	2
Peroxiredoxin-1	PRDX1_RAT	0	0	0	1
Peroxiredoxin-2	PRDX2_RAT	0	0	1	0

Peroxiredoxin-6	PRDX6_RAT	0	2	0	0
Phosphate carrier protein, mitochondrial	MPCP_RAT	6	2	0	0
Phosphatidate cytidylyltransferase 2	CDS2_RAT	0	0	2	2
Phosphatidylinositide phosphatase SAC1	SAC1_RAT	1	0	0	0
Phosphatidylinositol transfer protein alpha isoform	PIPNA_RAT	0	0	1	1
Phosphoglucomutase-1	PGM1_RAT	2	3	0	0
Phosphoglycerate kinase 1	PGK1_RAT	0	0	3	3
Phosphoglycerate mutase 1	PGAM1_RAT	0	1	1	2
Phospholipase A-2-activating protein	PLAP_RAT	0	0	1	2
Plasma membrane calcium-transporting ATPase 1	AT2B1_RAT	1	3	0	0
Plasma membrane calcium-transporting ATPase 2	AT2B2_RAT	0	1	0	0
Platelet-activating factor acetylhydrolase IB subunit alpha	LIS1_RAT	0	0	2	3
Polyadenylate-binding protein	PABP1_RAT	0	0	0	3
Polyubiquitin-B	UBB_RAT	0	0	3	3
Prenylcysteine oxidase	PCYOX_RAT	0	0	2	2
Prohibitin	PHB_RAT	0	0	1	1
Prohibitin-2	PHB2_RAT	4	3	2	3
Prolyl endopeptidase	PPCE_RAT	0	0	1	2
Proteasome subunit alpha type-1	PSA1_RAT	0	0	0	1
Proteasome subunit alpha type-2	PSA2_RAT	0	0	0	1
Proteasome subunit alpha type-3	PSA3_RAT	0	0	1	1

Proteasome subunit alpha type-4	PSA4_RAT	0	0	0	1
Proteasome subunit alpha type-6	PSA6_RAT	0	2	1	0
Proteasome subunit beta type-1	PSB1_RAT	0	1	0	1
Proteasome subunit beta type-4	PSB4_RAT	0	0	0	1
Proteasome subunit beta type-6	PSB6_RAT	0	0	0	1
Protein disulfide-isomerase A3	PDIA3_RAT	4	3	3	3
Protein disulfide-isomerase A6	PDIA6_RAT	0	0	3	3
Protein disulfide-isomerase	PDIA1_RAT	2	2	0	0
Protein DJ-1	PARK7_RAT	0	0	1	1
Protein IMPACT	IMPCT_RAT	0	0	1	2
Protein kinase C gamma type	KPCG_RAT	0	0	2	3
Protein phosphatase 1 regulatory subunit 7	PP1R7_RAT	0	0	0	2
Protein phosphatase 1E	PPM1E_RAT	0	0	1	0
Protein phosphatase 1H	PPM1H_RAT	0	0	1	0
Pyruvate carboxylase, mitochondrial	PYC_RAT	0	0	2	2
Pyruvate dehydrogenase E1 component subunit alpha, somatic form, mitochondrial	ODPA_RAT	2	3	2	2
Pyruvate dehydrogenase E1 component subunit beta, mitochondrial	ODPB_RAT	3	2	2	1
Pyruvate kinase PKM	KPYM_RAT	0	0	2	3
Rab GDP dissociation inhibitor alpha	GDIA_RAT	6	4	3	3
Rab GDP dissociation inhibitor beta	GDIB_RAT	4	4	2	3

Ras-related C3 botulinum toxin substrate 1	RAC1_RAT	0	0	1	0
Ras-related protein Rab-11A	RB11A_RAT	0	0	1	0
Ras-related protein Rab-11B	RB11B_RAT	0	0	0	1
Ras-related protein Rab-1A	RAB1A_RAT	0	0	1	1
Ras-related protein Rab-3A	RAB3A_RAT	0	0	2	1
Ras-related protein Rab-3B	RAB3B_RAT	0	0	0	1
Ras-related protein Rab-3C	RAB3C_RAT	0	1	0	0
Ras-related protein Rab-6A	RAB6A_RAT	0	0	0	1
Ras-related protein Rab-7a	RAB7A_RAT	0	0	1	1
Ras-related protein Ral-A	RALA_RAT	0	1	1	1
Receptor-type tyrosine-protein phosphatase alpha	PTPRA_RAT	0	0	0	1
Reticulon-4	RTN4_RAT	1	0	0	0
Rho GDP-dissociation inhibitor 1	GDIR1_RAT	0	1	1	2
Rho-related GTP-binding protein RhoB	RHOB_RAT	0	1	0	0
Ribose-phosphate pyrophosphokinase 1	PRPS1_RAT	0	0	0	1
Sarcoplasmic/endoplasmic reticulum calcium ATPase 1	AT2A1_RAT	0	1	0	0
Secernin-1	SCRN1_RAT	0	0	1	2
Septin-11	SEP11_RAT	3	2	0	0
Septin-2	SEPT2_RAT	0	0	0	2
Septin-7	SEPT7_RAT	0	1	0	0
Serine/threonine-protein kinase BRSK1	BRSK1_RAT	0	0	1	0

Serine/threonine-protein kinase DCLK1	DCLK1_RAT	0	0	2	3
Serine/threonine-protein phosphatase 2A 55 kDa regulatory subunit B alpha isoform	2ABA_RAT	0	0	1	2
Serine/threonine-protein phosphatase 2A catalytic subunit alpha isoform	PP2AA_RAT	0	0	1	3
Serine/threonine-protein phosphatase 2A catalytic subunit beta isoform	PP2AB_RAT	0	0	1	0
Serine/threonine-protein phosphatase 2B catalytic subunit alpha isoform	PP2BA_RAT	4	3	1	3
Serine/threonine-protein phosphatase 2B catalytic subunit beta isoform	PP2BB_RAT	0	0	2	2
Serine/threonine-protein phosphatase PP1-alpha catalytic subunit	PP1A_RAT	0	0	1	2
Serine/threonine-protein phosphatase PP1-beta catalytic subunit	PP1B_RAT	0	0	0	1
Serine/threonine-protein phosphatase PP1-gamma catalytic subunit	PP1G_RAT	0	1	1	1
Serpin H1	SERPH_RAT	0	0	0	2
S-formylglutathione hydrolase	ESTD_RAT	0	0	1	2
Short-chain specific acyl-CoA dehydrogenase, mitochondrial	ACADS_RAT	0	0	1	3
Sideroflexin-1	SFXN1_RAT	5	2	3	2
Sideroflexin-3	SFXN3_RAT	0	0	2	3
Sodium- and chloride-dependent GABA transporter 1	SC6A1_RAT	0	0	0	2
Sodium/potassium-transporting ATPase subunit alpha-1	AT1A1_RAT	0	0	3	3

Sodium/potassium-transporting ATPase subunit alpha-2	AT1A2_RAT	2	3	0	0
Sodium/potassium-transporting ATPase subunit alpha-3	AT1A3_RAT	6	4	3	3
Sodium/potassium-transporting ATPase subunit beta-1	AT1B1_RAT	5	4	3	3
Somatotropin	SOMA_RAT	0	1	0	0
Sorting nexin-1	SNX1_RAT	1	0	0	0
Spectrin alpha chain, non-erythrocytic 1	SPTN1_RAT	0	0	1	1
Spliceosome RNA helicase Ddx39b	DX39B_RAT	3	3	0	1
Staphylococcal nuclease domain-containing protein 1	SND1_RAT	0	0	1	1
STE20-like serine/threonine-protein kinase	SLK_RAT	0	1	0	0
Stress-70 protein, mitochondrial	GRP75_RAT	3	3	0	0
Stress-induced-phosphoprotein 1	STIP1_RAT	1	3	0	0
Succinate dehydrogenase [ubiquinone] flavoprotein subunit, mitochondrial	DHSA_RAT	2	2	0	0
Succinate dehydrogenase [ubiquinone] iron-sulfur subunit, mitochondrial	DHSB_RAT	0	1	0	0
Succinyl-CoA ligase [ADP/GDP-forming] subunit alpha, mitochondrial	SUCA_RAT	0	0	2	1
Succinyl-CoA:3-ketoacid coenzyme A transferase 1, mitochondrial	SCOT1_RAT	0	0	3	3
Synapsin-1	SYN1_RAT	4	4	0	0
Synapsin-2	SYN2_RAT	3	4	0	0
Synaptic vesicle glycoprotein 2A	SV2A_RAT	2	3	3	3
Synaptic vesicle glycoprotein 2B	SV2B_RAT	0	0	0	1

Synaptic vesicle membrane protein VAT-1 homolog	VAT1_RAT	2	1	1	0
Synaptojanin-1	SYNJ1_RAT	0	1	0	0
Synaptophysin	SYPH_RAT	3	4	1	2
Synaptotagmin-1	SYT1_RAT	5	4	3	3
Syntaxin-1A	STX1A_RAT	0	0	0	1
Syntaxin-1B	STX1B_RAT	0	0	3	2
T-complex protein 1 subunit alpha	TCPA_RAT	2	4	2	3
T-complex protein 1 subunit beta	TCPB_RAT	0	0	3	3
T-complex protein 1 subunit delta	TCPD_RAT	1	3	3	3
T-complex protein 1 subunit epsilon	TCPE_RAT	2	3	2	2
T-complex protein 1 subunit gamma	TCPG_RAT	5	3	2	3
Thimet oligopeptidase	THOP1_RAT	0	0	1	0
Threonine--tRNA ligase, cytoplasmic	SYTC_RAT	0	0	1	0
Transaldolase	TALDO_RAT	4	2	1	3
Transcriptional activator protein Pur-alpha (Fragments)	PURA_RAT	0	0	1	2
Transcriptional activator protein Pur-beta	PURB_RAT	0	0	3	2
Transitional endoplasmic reticulum ATPase	TERA_RAT	4	4	3	3
Transketolase	TKT_RAT	3	4	2	3
Transmembrane emp24 domain-containing protein 9	TMED9_RAT	0	0	0	1
Trifunctional enzyme subunit alpha, mitochondrial	ECHA_RAT	0	0	0	2
Trifunctional enzyme subunit beta, mitochondrial	ECHB_RAT	1	3	0	1

Triosephosphate isomerase	TPIS_RAT	1	2	1	1
Tripartite motif-containing protein 2	TRIM2_RAT	0	0	0	1
Tripeptidyl-peptidase 2	TPP2_RAT	0	1	0	2
Tropomyosin alpha-1 chain	TPM1_RAT	0	1	0	0
Tropomyosin alpha-3 chain	TPM3_RAT	0	1	0	0
Tropomyosin beta chain	TPM2_RAT	0	1	0	0
Troponin T, fast skeletal muscle	TNNT3_RAT	0	1	0	0
Tubulin alpha-1A chain	TBA1A_RAT	0	0	5	3
Tubulin alpha-1B chain	TBA1B_RAT	0	0	1	0
Tubulin alpha-4A chain	TBA4A_RAT	0	0	1	3
Tubulin beta-2A chain	TBB2A_RAT	0	0	4	5
Tubulin beta-2B chain	TBB2B_RAT	0	1	3	3
Tubulin beta-3 chain	TBB3_RAT	0	0	3	3
Tyrosine-protein kinase Fyn	FYN_RAT	0	0	2	2
Tyrosine--tRNA ligase, cytoplasmic	SYYC_RAT	0	0	1	0
Ubiquitin carboxyl-terminal hydrolase isozyme L1	UCHL1_RAT	1	2	1	1
Ubiquitin thioesterase OTUB1	OTUB1_RAT	2	2	1	2
Ubiquitin-like modifier-activating enzyme 1	UBA1_RAT	5	4	3	3
UDP-glucose:glycoprotein glucosyltransferase 1	UGGG1_RAT	0	0	0	1
Unconventional myosin-Va	MYO5A_RAT	0	1	3	3
Valyl-tRNA synthetase	SYVC_RAT	3	3	0	0

Vesicular glutamate transporter 1	VGLU1_RAT	1	0	0	0
Vigilin	VIGLN_RAT	0	1	0	0
Voltage-dependent anion-selective channel protein 1	VDAC1_RAT	5	4	3	3
Voltage-dependent anion-selective channel protein 2	VDAC2_RAT	4	3	3	3
Voltage-dependent anion-selective channel protein 3	VDAC3_RAT	1	3	1	2
Voltage-dependent calcium channel subunit alpha-2/delta-1	CA2D1_RAT	0	0	0	1
V-type proton ATPase subunit B, brain isoform	VATB2_RAT	3	3	3	3
V-type proton ATPase subunit C 1	VATC1_RAT	0	0	0	2
V-type proton ATPase subunit E 1	VATE1_RAT	4	3	0	0
WD repeat-containing protein 1	WDR1_RAT	3	3	2	3
Wiskott-Aldrich syndrome protein family member 1	WASF1_RAT	0	0	1	2
Total: 447					

Supplemental Table 2: Protein list of all identified proteins. The columns indicate the protein name, the UniProt accession number and the number of detections of the protein in hippocampal and cortical neurons in control or NMDA-stimulated cultures.

SUPPLEMENTAL TABLE 3

	**corr. p-Value**	
**Biological Process**	**no stimulus**	**NMDA**
glycolysis	4.99E-14	1.62E-06
tricarboxylic acid cycle	1.29E-13	2.37E-06
protein folding	3.87E-12	6.18E-08

GTP catabolic process	4.12E-11	2.91E-07
ATP catabolic process	2.50E-10	7.44E-08
2-oxoglutarate metabolic process	1.03E-09	1.37E-05
ATP biosynthetic process	4.92E-09	5.80E-07
carbohydrate metabolic process	4.16E-08	1.60E-04
synaptic transmission	8.32E-08	3.72E-05
oxaloacetate metabolic process	1.69E-07	1.62E-03
isocitrate metabolic process	1.77E-07	8.57E-03
NADH metabolic process	3.31E-06	1.59E-02
protein phosphorylation	1.23E-04	3.39E-06
cell cycle	7.13E-06	4.49E-06
cell division	6.79E-05	4.90E-06
pentose-phosphate shunt	6.04E-06	1.87E-02
response to drug	7.16E-06	2.12E-04
gluconeogenesis	8.08E-06	5.64E-03
protein export from nucleus	4.94E-05	1.37E-05
protein autophosphorylation	1.94E-04	1.53E-05
intracellular protein transport	1.66E-05	1.71E-04
response to organic cyclic compound	1.69E-05	1.19E-03
neurotransmitter transport	1.70E-05	4.52E-04
transport	1.74E-05	8.50E-04
small GTPase mediated signal transduction	2.05E-05	3.30E-03
response to stress	2.23E-05	5.83E-05
brain development	2.47E-05	2.86E-05
neuromuscular process controlling balance	1.23E-04	2.98E-05
response to toxin	2.82E-04	3.63E-05
cellular carbohydrate metabolic process	3.71E-05	3.55E-02
response to hypoxia	4.13E-05	2.17E-04

protein transport	4.94E-05	1.30E-04
glutathione metabolic process	5.17E-05	1.62E-03
endocytosis	6.39E-05	6.01E-04
translational elongation	7.97E-05	4.74E-03
ionotropic glutamate receptor signaling pathway	1.05E-04	1.18E-03
metabolic process	1.12E-04	9.81E-03
neuron projection development	1.14E-04	4.18E-03
regulation of protein localization	1.15E-04	3.04E-04
axonogenesis	1.23E-04	1.49E-04
nervous system development	1.31E-04	2.73E-03
protein dephosphorylation	1.35E-04	4.40E-03
aging	4.36E-04	2.03E-04
vesicle-mediated transport	2.16E-04	2.15E-03
NAD metabolic process	2.18E-04	
regulation of the force of heart contraction	6.85E-04	2.22E-04
response to reactive oxygen species	6.85E-04	2.22E-04
muscle filament sliding	4.94E-04	2.36E-04
regulation of long-term neuronal synaptic plasticity	3.18E-04	9.27E-03
microtubule-based process	1.36E-03	5.54E-04
cellular response to growth factor stimulus	2.22E-03	5.69E-04
neural retina development	1.21E-03	6.00E-04
regulation of stress-activated MAPK cascade	1.21E-03	6.00E-04
microtubule-based movement	6.44E-04	6.21E-03
proteolysis	6.89E-04	2.92E-02
response to cocaine	7.02E-04	1.74E-03
regulation of heart contraction	1.82E-03	7.24E-04
response to nutrient	1.04E-03	8.30E-04
acetyl-CoA biosynthetic process from pyruvate	8.36E-04	

pyrimidine base catabolic process	8.36E-04	
negative regulation of microtubule depolymerization	8.54E-04	3.68E-03
calcium ion transport	8.66E-04	4.63E-03
protein polymerization	8.88E-04	2.06E-03
regulation of exocytosis	1.01E-03	4.24E-03
organ regeneration	4.14E-03	1.13E-03
response to cytokine stimulus	1.13E-03	1.21E-03
actin filament capping	2.21E-03	1.18E-03
citrate metabolic process	1.21E-03	1.08E-02
malate metabolic process	1.21E-03	
regulation of protein transport	1.21E-03	
response to electrical stimulus	2.86E-03	1.21E-03
response to morphine	2.86E-03	1.21E-03
protein homotetramerization	3.44E-03	1.23E-03
response to heat	1.24E-03	1.69E-03
cellular calcium ion homeostasis	1.25E-03	1.70E-03
response to ethanol	1.84E-03	1.33E-03
calcium ion transmembrane transport	3.32E-03	1.33E-03
positive regulation of anti-apoptosis	1.34E-03	2.84E-03
mitosis	5.86E-03	1.46E-03
caveolin-mediated endocytosis	2.10E-03	1.48E-03
regulation of interferon-gamma-mediated signaling pathway	2.10E-03	1.48E-03
regulation of type I interferon-mediated signaling pathway	2.10E-03	1.48E-03
muscle contraction	4.10E-03	1.54E-03
phosphorylation	4.10E-03	1.54E-03
regulation of multicellular organism growth	4.10E-03	1.54E-03
response to estrogen stimulus	1.66E-03	1.55E-03
myoblast fusion	3.42E-03	1.62E-03

purine nucleotide biosynthetic process	3.42E-03	1.62E-03
liver development	1.82E-03	1.69E-03
cation transport	6.40E-03	1.70E-03
anti-apoptosis	1.73E-03	1.85E-02
multicellular organismal development	1.80E-03	1.74E-03
bone resorption	1.82E-03	4.54E-02
cholesterol biosynthetic process	1.82E-03	4.54E-02
glucose metabolic process	1.83E-03	3.62E-03
learning or memory	1.83E-03	3.62E-03
regulation of cell shape	1.84E-03	
insulin-like growth factor receptor signaling pathway	4.24E-03	1.86E-03
response to growth hormone stimulus	4.24E-03	1.86E-03
response to calcium ion	1.99E-03	2.71E-03
learning	6.21E-03	2.06E-03
microtubule cytoskeleton organization	2.08E-03	4.25E-03
fatty acid metabolic process	2.09E-03	2.80E-03
cellular response to oxidative stress	2.10E-03	6.79E-03
aldehyde catabolic process	2.10E-03	4.56E-02
glyceraldehyde-3-phosphate metabolic process	2.10E-03	
glyoxylate cycle	2.10E-03	4.56E-02
ribose phosphate biosynthetic process	2.10E-03	4.56E-02
uropod organization	2.10E-03	4.56E-02
actin cytoskeleton organization	2.11E-03	3.93E-02
ATP metabolic process	2.13E-03	
electron transport chain	2.23E-03	4.51E-03
glycogen metabolic process	2.50E-03	8.53E-03
intracellular protein kinase cascade	2.68E-03	1.73E-02
labyrinthine layer blood vessel development	6.19E-03	2.79E-03

MAPK import into nucleus	4.91E-03	2.84E-03
chaperone-mediated protein complex assembly	4.91E-03	2.84E-03
ketone body catabolic process	4.91E-03	2.84E-03
pentose-phosphate shunt, oxidative branch	4.91E-03	2.84E-03
positive regulation of dopamine metabolic process	4.91E-03	2.84E-03
regulation of muscle contraction	7.37E-03	3.12E-03
forebrain development	1.15E-02	3.12E-03
oxidation-reduction process	3.31E-03	5.54E-03
neuron migration	3.46E-03	4.18E-03
sarcomere organization	8.98E-03	4.24E-03
microtubule organizing center organization	7.91E-03	4.38E-03
protein import into mitochondrial outer membrane	7.91E-03	4.38E-03
protein localization to microtubule	7.91E-03	4.38E-03
regulation of ATPase activity	7.91E-03	4.38E-03
regulation of Golgi inheritance	7.91E-03	4.38E-03
regulation of early endosome to late endosome transport	7.91E-03	4.38E-03
regulation of protein export from nucleus	7.91E-03	4.38E-03
regulation of striated muscle contraction	7.91E-03	4.38E-03
cell adhesion	1.41E-02	4.43E-03
response to organic substance	6.34E-03	4.51E-03
endoplasmic reticulum tubular network organization	4.91E-03	
gamma-aminobutyric acid metabolic process	4.91E-03	
response to food	1.15E-02	4.96E-03
response to hormone stimulus	5.16E-03	5.41E-03
protein heterooligomerization	2.14E-02	5.83E-03
response to estradiol stimulus	8.37E-03	5.84E-03
chaperone mediated protein folding requiring cofactor	6.19E-03	
response to amphetamine	6.21E-03	1.60E-02

Rab protein signal transduction	1.14E-02	6.22E-03
cell morphogenesis involved in differentiation	1.14E-02	6.22E-03
fructose 1,6-bisphosphate metabolic process	1.14E-02	6.22E-03
negative regulation of necrotic cell death	1.14E-02	6.22E-03
succinyl-CoA metabolic process	1.14E-02	6.22E-03
negative regulation of apoptotic process	8.34E-03	6.23E-03
epithelial to mesenchymal transition	1.50E-02	6.26E-03
response to organic nitrogen	6.40E-03	1.01E-02
ventricular cardiac muscle tissue morphogenesis	1.62E-02	6.79E-03
peptidyl-serine phosphorylation	2.13E-02	7.18E-03
regulation of protein catabolic process	7.37E-03	3.55E-02
lipid metabolic process	1.10E-02	7.80E-03
cellular response to inorganic substance	7.91E-03	
glutamate biosynthetic process	7.91E-03	
neurotransmitter biosynthetic process	7.91E-03	
pentose-phosphate shunt. non-oxidative branch	7.91E-03	
adult locomotory behavior	8.09E-03	2.00E-02
visual learning	8.25E-03	1.91E-02
cell differentiation	8.34E-03	3.66E-02
lipopolysaccharide-mediated signaling pathway	2.03E-02	8.53E-03
apical protein localization	1.54E-02	8.57E-03
synaptic vesicle maturation	1.54E-02	8.57E-03
synaptic vesicle transport	1.54E-02	8.57E-03
calcium ion-dependent exocytosis	8.60E-03	3.90E-02
establishment of cell polarity	8.60E-03	3.90E-02
ATP hydrolysis coupled proton transport	8.82E-03	
cytokinesis	8.82E-03	
regulation of cell migration	8.82E-03	

cell migration	8.85E-03	
protein homooligomerization	8.85E-03	
hydrogen peroxide catabolic process	8.98E-03	3.22E-02
response to vitamin E	8.98E-03	3.22E-02

Supplemental Table 3. Filtered Genecodis results: Single enrichment analyses were performed with Genecodis on the SNO-proteome data of the NMDA stimulated and unstimulated rats. The resulting lists were merged via their Gene Ontology Terms (biological processes). A cut-off for the adjusted p-values was set at 0.01 and p-values cells were filled grey, if and only if the p-values were at least $1x10^{-1}$ apart, marking the group with potentially more proteins dedicated to the biological process.
