# Supplementary figures and images for: eNOS-dependent S-nitrosylation of the NF-κB subunit p65 has neuroprotective effects
Source: Cell Death Dis. 2021 Jan 4;12(1):4. doi: 10.1038/s41419-020-03338-4 (PMC7790835; doi:10.1038/s41419-020-03338-4)

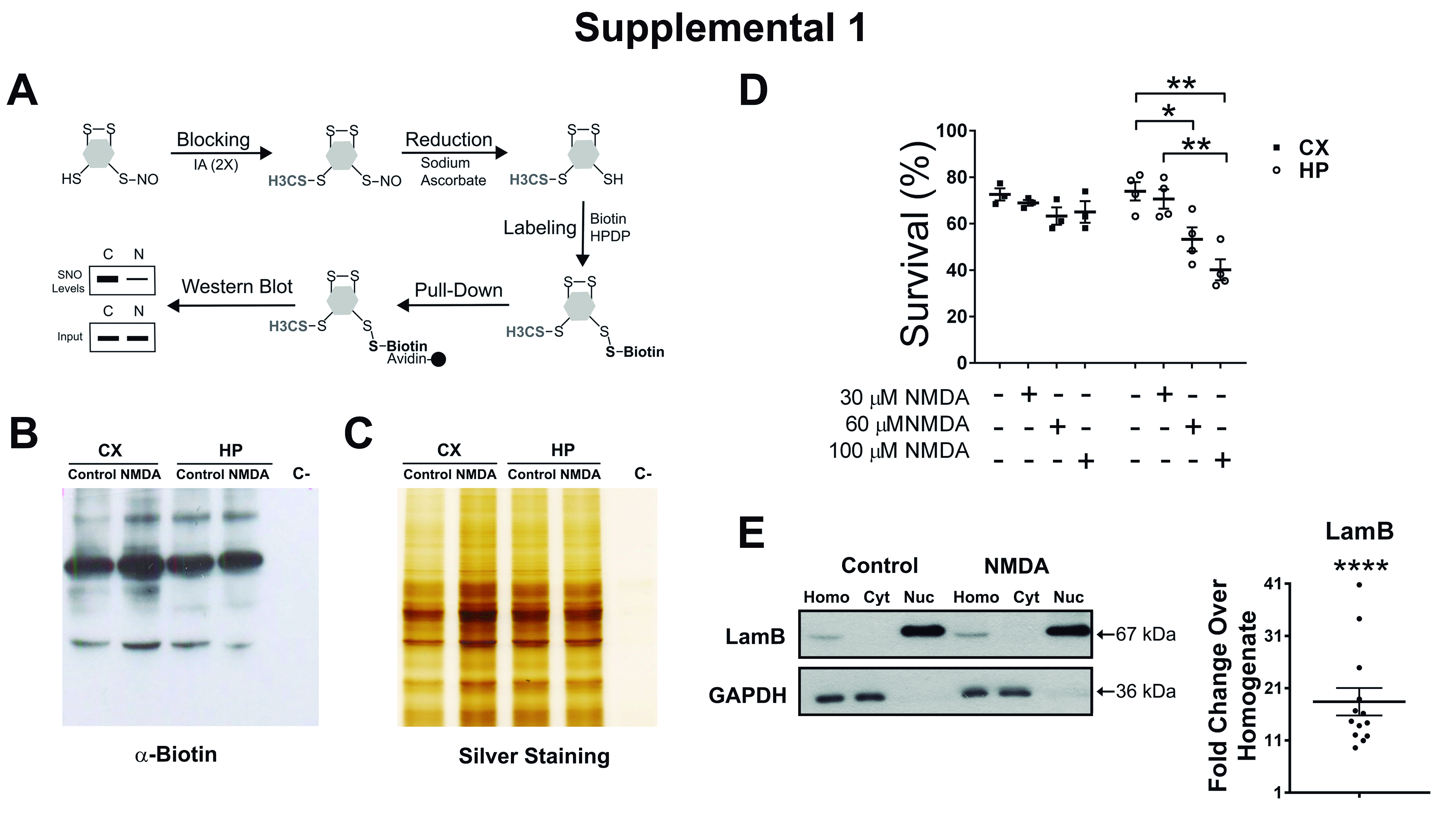

Supplement: Supplementary file 2 — Supplemental Figure S1 [file 41419_2020_3338_MOESM2_ESM.tif]

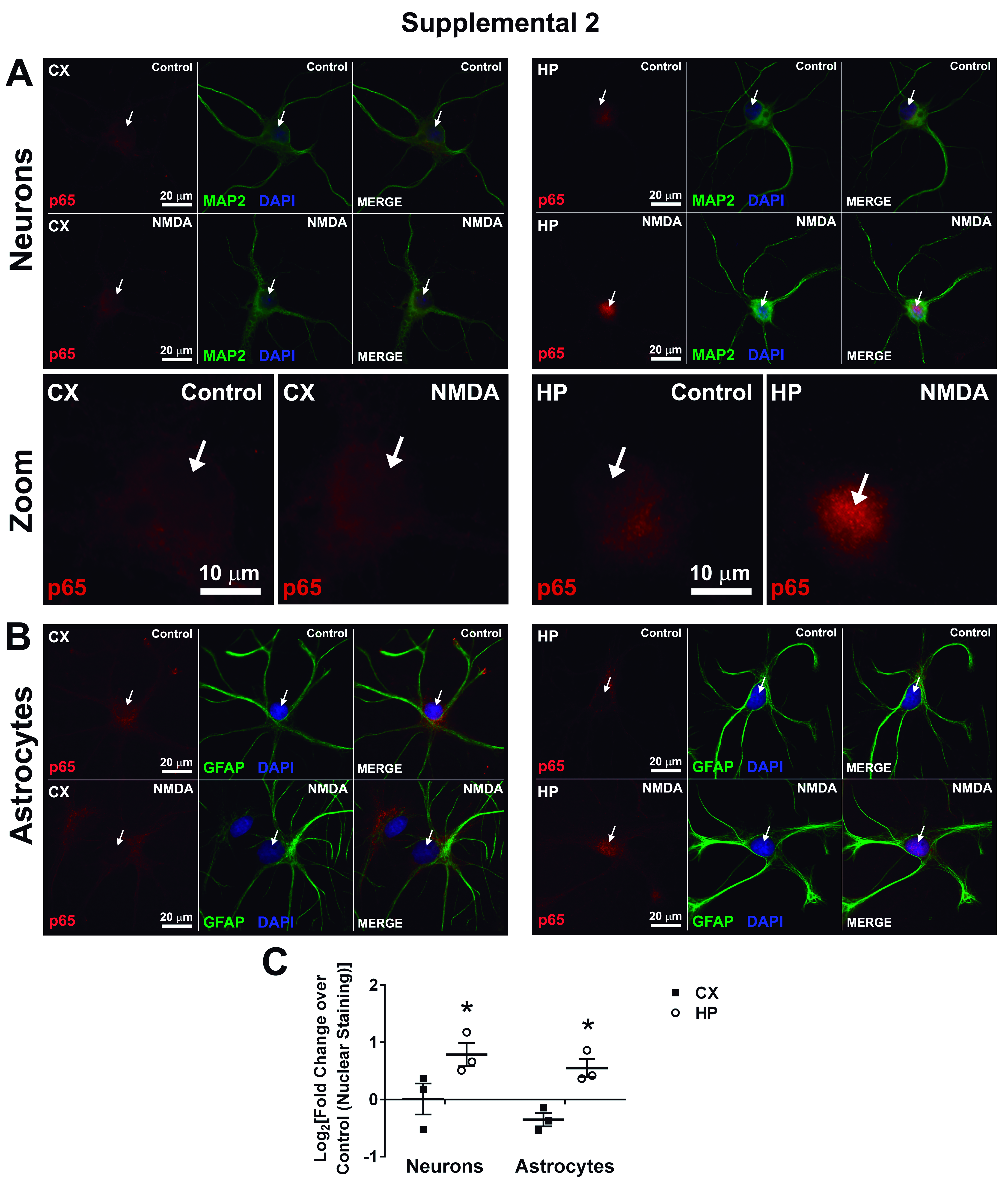

Supplement: Supplementary file 3 — Supplemental Figure S2 [file 41419_2020_3338_MOESM3_ESM.tif]

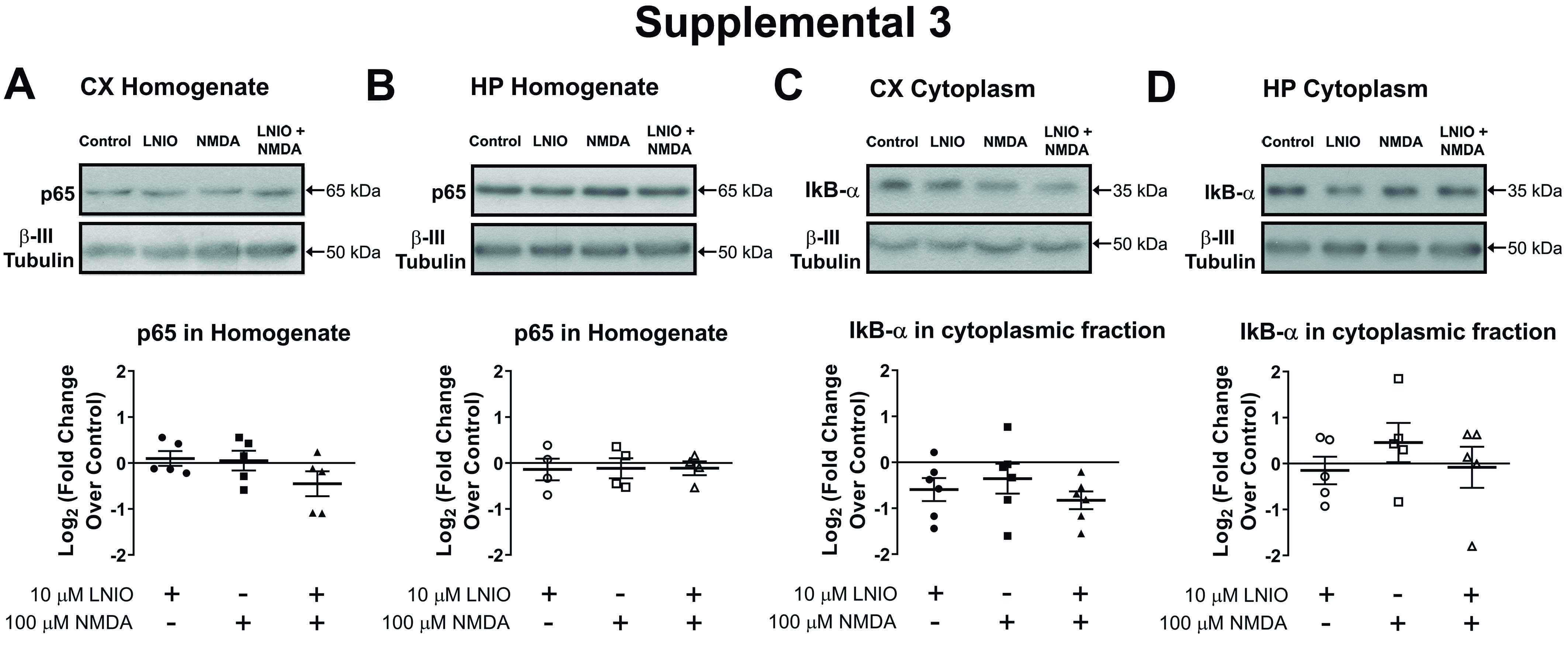

Supplement: Supplementary file 4 — Supplemental Figure S3 [file 41419_2020_3338_MOESM4_ESM.tif]

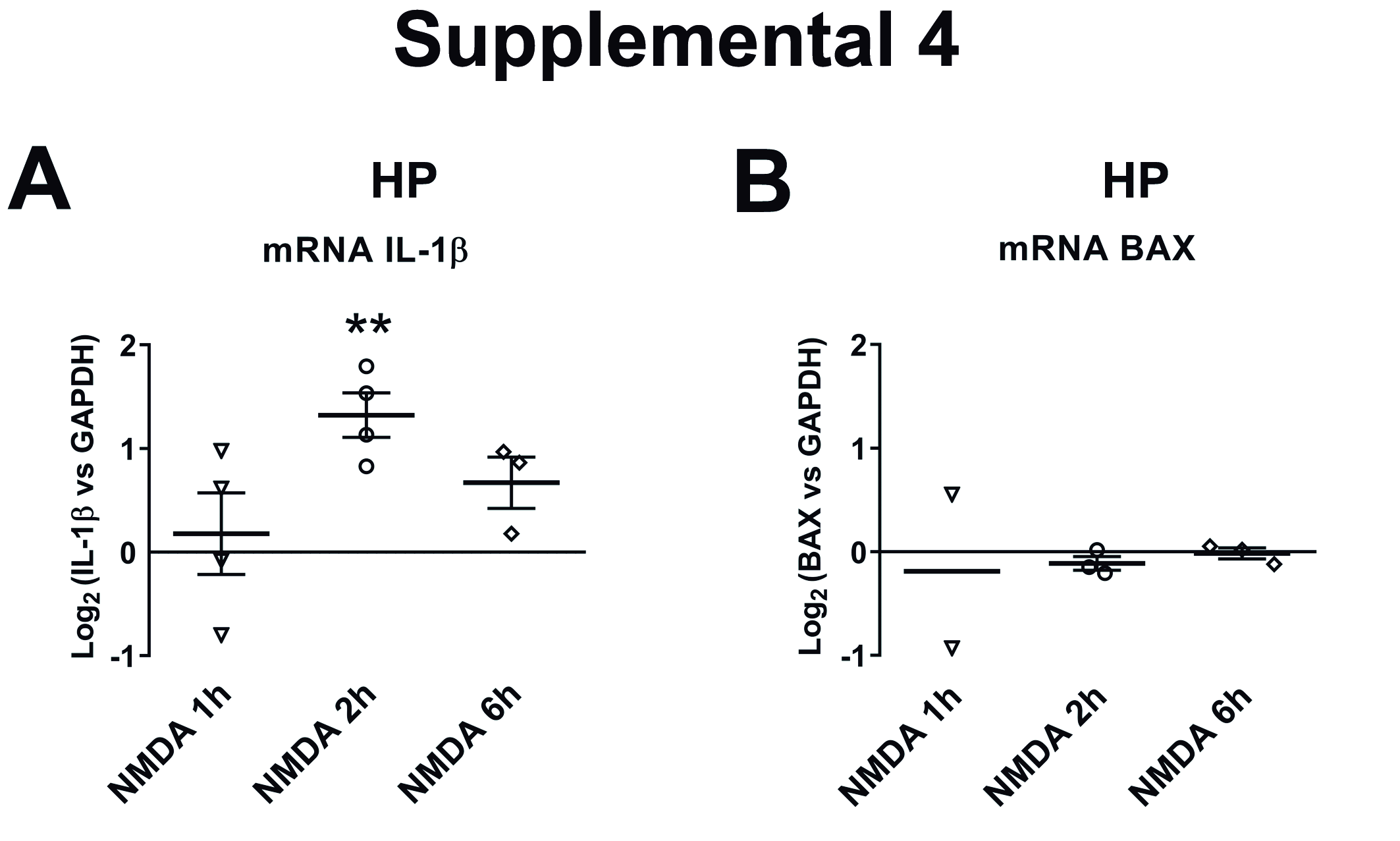

Supplement: Supplementary file 5 — Supplemental Figure S4 [file 41419_2020_3338_MOESM5_ESM.tif]

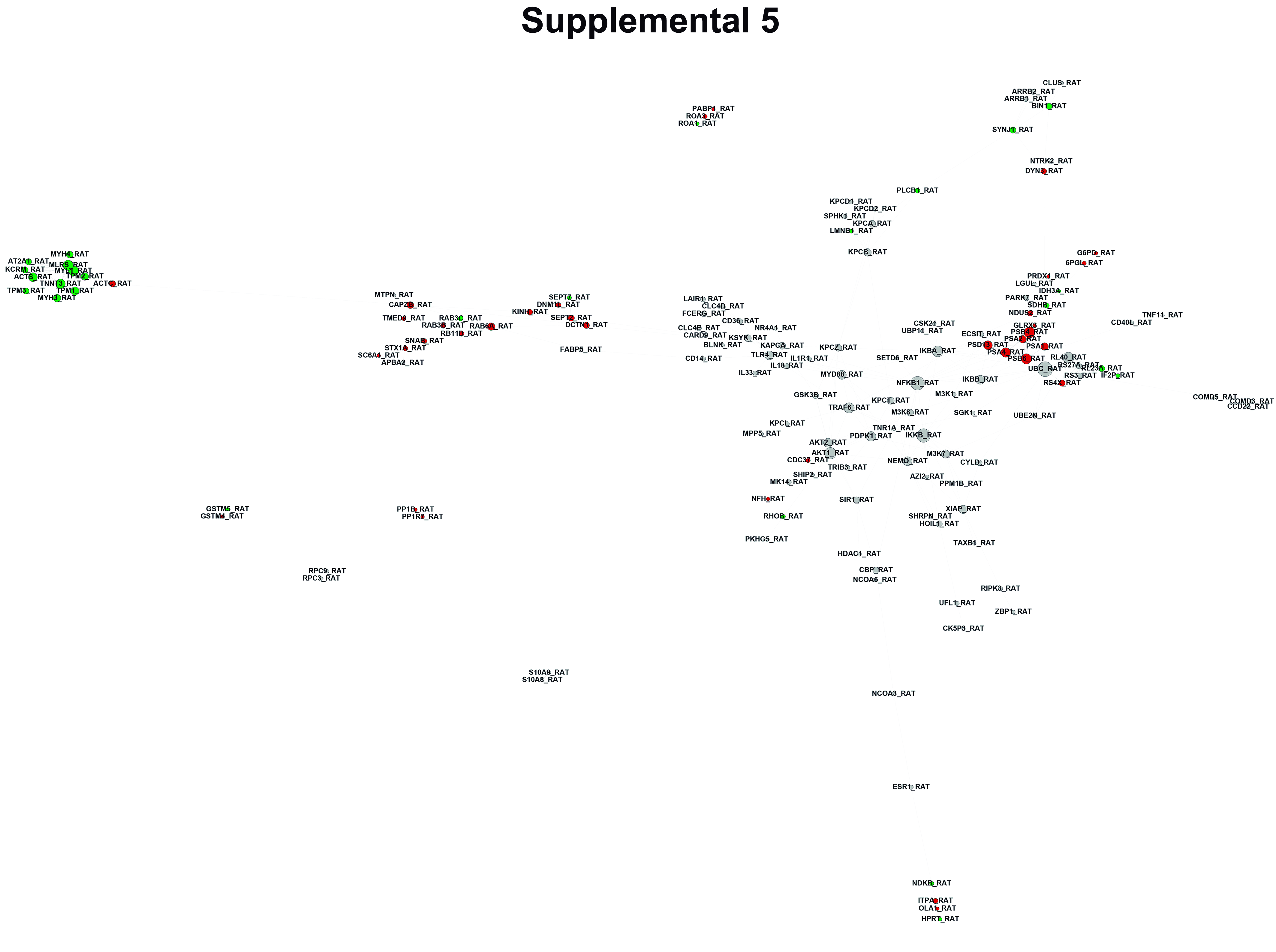

Supplement: Supplementary file 6 — Supplemental Figure S5 [file 41419_2020_3338_MOESM6_ESM.tif]
